# IPA 2009 International Meeting (IPA Rio)

**Published:** 2009

The Brazilian Association of Geriatric Neuropsychiatry (ABNPG) and the International Psychogeriatric Association (IPA) are proud to present in the following pages the poster, free communications, and presentation abstracts shown during the International Meeting of the IPA and Congress of the ABNPG in Rio de Janeiro, May, 4^th^–7^th^, 2009.

The meeting congregated more than 480 participants from 30 countries, who keenly discussed various subjects and aspects on the official theme of the meeting, Brain Aging and Quality of Life. This diversity of subjects and the quality of the presentations can be clearly observed in the abstracts. By having them published in **Dementia & Neuropsychologia**, the organizing committee of the meeting can affirm that the task of improving the communication among the clinicians and research groups from various countries will be attained in addition to the lively debates and face to face discussions which took place.

We wish to thank all who presented their work in the meeting. This was a key factor for its success.

Warm Regards,

Jerson Laks, João Carlos Machado, Paulo Caramelli

## ABSTRACT – 1

**Contemporary issues on depression in dementia**

Engedal K 

Norwegian Centre for Ageing and Health, Oslo University Hospital, Oslo, Norway.

The co-existence of poor mood and memory have been known for hundred of years, but is not well understood. The prevalence rate of depression in demented patients varies between studies from zero to 86%, and is in average 20% for a depressive disorder and about 50% for any depressive symptomatology. The variability in prevalence rates is probably due to methodological differences between studies like definitions of depression, assessment methods used, sample selection and clinical manifestation. Depression in dementia may fluctuate over time, but tend in a substantial number of patients to be of chronic, but not of progressive nature. Compared to elderly depressed patients without dementia the persistence rate (chronicity) is higher and the recovery rate lower. Further, the prognosis of depression in dementia with regard to quality of life, autonomy, function in activities of daily living, morbidity and mortality is poorer compared to the prognosis of patients suffering from depression without dementia. The moderate increase in mortality conferred by the presence of depression appears though in most studies (but not all) to be related to co-morbid physical disorders. The causal relationship between depression and dementia, especially Alzheimer’s disease (AD) remains unclear. Both psychological and biological explanation for the relationship has been suggested. Several studies have reported a higher prevalence rate of depression in mild compared to severe dementia, and suggested that awareness of dementia may trigger depression because of social stress, loss of autonomy, dignity and self-esteem. Other studies have suggested that depression is more prevalent in severe Alzheimer’s dementia. Some neuropathological studies have found changes in adrenergic cells in locus coeruleus in AD patients at post-mortem examination, and put forward a hypothesis that due to progressive neuronal degeneration of monoaminergic brainstem nuclei and loss of adrenergic cells in locus coeruleus depression should be more prevalent as AD progresses. Recently a study could not confirm this hypothesis. Besides, a systematic review from 2007 concluded that there is no association between severity of Alzheimer’s disease and prevalence of depression or depressive symptoms. However, depression and AD share a range of risk factors and findings: hypertension, diabetes, obesity, hyperhomocysteinemia, smoking, lack of exercise, changes in cytokines (IL-1, IL-6, TNF-alpha), HPA axis activation and structural brain changes such as medial temporal lobe atrophy and white matter lesions. It is therefore not unlikely that the two disorders share putative underlying mechanisms, but we do not know whether this observed relationship merely reflect that both conditions have common risk factors. One hypothesis is that neuritic plaqus and neurofibrillary tangles underlie depression in AD. Up to date no study could confirm this hypothesis. Other studies have been published suggesting that depression could be a risk factor for AD. Recently, a neuropathological study came to another conclusion and demonstrated that depression could be a risk factor for clinical AD in the presence of AD pathology, but not a risk factor for AD pathology. Another study came to the conclusion that the association between depression and AD is independent of AD pathology. It can be summarised that there are several associations between depression and Alzheimer’s dementia, but we do not understand the mechanisms for these associations. Some studies report a different constellation of depressive symptoms in patients with dementia compared to those without dementia. Demented patients do not tend to express mood feelings like sadness, hopelessness and guilt, but to report anxiety and they are more often apathetic and have more often delusions and hallucinations. There might be several explanations for this observation. Firstly, symptoms of depression and dementia overlap and symptoms of dementia can be interpreted as signs of depression. Secondly, due to aphasia and semantic memory problems patients with severe degree of dementia have problems in expressing feelings and it is therefore difficult to know whether such patients have a feeling of sadness or even suicidal thoughts. To interpret their body language is not easy either. However, some studies have reported that a different symptom constellation is only present in cases where mood symptoms lack. Possibly, various forms of depression exist in dementia, which should lead to different treatments. Attempts have been made to define a specific form of depression in AD, such as the ‘Provisional diagnostic criteria for depression of AD’. The criteria have not been validated, and it is therefore difficult to judge whether or not these criteria is useful for the understanding of depression in dementia. Various recommendations exist for drug treatment of depression in dementia, but according to systematic reviews the evidence for an effect of antidepressants is poor. The Cochrane review of 2002, including seven studies of sufficient quality and the review by Thompson et al. from 2007, including only five studies both conclude that the evidence for effect of antidepressants is poor compared to the effect seen in elderly without dementia. Both reviews did not report any differences in effect between drugs. No withdrawal study of antidepressants has been carried out.

***Conclusion:*** Depression in dementia is prevalent, but the understanding of the relationship between the two conditions is poor. The rather modest effect of antidepressants may reflect that depression in dementia is a different condition compared to depression observed in elderly no-demented subjects.

## ABSTRACT – 2

**The relationship of white matter lesions with mood and cognition**

Pantoni L

Department of Neurological and Psychiatric Sciences, University of Florence, Firenze, Italy.

Age-related white matter changes (ARWMC), also called leukoaraiosis, are frequently found by neuroimaging in elderly patients with various clinical disturbances. The clinical relevance of ARWMC has been extensively studied and better defined over the last decade. It has become quite clear that mild degrees of ARWMC are almost invariably found after the age of 65 and should be considered as part of the aging process. On the other hand, moderate-to-severe degrees of ARWMC are associated with cognitive, mood, gait and urinary disturbances interfering with every day activities. A considerable number of studies have found ARWMC to be associated with a certain type of cognitive disturbances. In patients with subcortical vascular changes such as ARWMC cognitive deficits are typically found in cognitive tasks related to frontal-subcortical circuits integrity such as executive functions, attention, speed, and set-shifting rather than in memory tasks. When severe, these cognitive deficits are part of the picture of subcortical vascular dementia and indeed ARWMC have been found associated with an increased risk of dementia and of the transition from normal status to Mild Cognitive Impairment. Patients who have moderate-to- severe ARWMC also have more severe depressive symptoms in comparison with patients with no or mild ARWMC. Often these symptoms coexist with more or less severe cognitive changes. Not only ARWMC are associated with depressive symptoms but also they predict their development in follow-up studies. Finally, ARWMC are among the neuroimaging correlates of vascular depression a concept recently introduced to describe the occurrence of depression in elderly with underlying vascular diseases. It is likely that the cognitive deficits and mood symptoms associated with ARWMC are among the explanations of the recently shown effect of ARWMC on the transition from functional autonomy to disability in the elderly.

## ABSTRACT – 3

Improving care for persons with AD: the Seattle protocols - Advances in evidence-based nonpharmacological treatment

Teri L

University of Washington, Seattle, WA, USA.

Behavioral problems are prevalent among persons with AD and significantly impair their health, well-being, and quality of life; our ability to provide effective care; and the life of their caregivers. Despite this, effective pharmacological treatments are of questionable utility. Indeed, experts and guidelines from various professional associations agree that medications are “the last resort” and nonpharmacological strategies should be implemented first. This presentation will provide an overview and summary data on the Seattle protocols, a series of systematic randomized controlled clinical trials designed to reduce behavioral problems in persons with Alzheimer’s disease via education, support and skill-training of their caregivers. The goal of these studies is to establish conceptually sound and clinically relevant treatment approaches and to evaluate their effectiveness along the diverse continuum of environments in which older adults reside and receive care (e. g. , private homes, retirement communities, assisted-living residences, adult family homes, and skilled nursing facilities). Promising findings thus far include clinically and statistically significant: decreases in patient depression, anxiety, and associated behavioral problems; improvements in patient quality of life; decreases in caregiver depression and burden; and improvements in staff satisfaction and burden. It is hoped that these data will help inform guidelines for evidence-based treatment of persons with Alzheimer’s disease in order to effectively improve their care.

## ABSTRACT – 4

**Frontal executive control in subcortical ischemic white matter disease**

Alves GS, Alves CE, Sudo F, Valente LE, Lanna ME, Laks J, Lanna ME, Moreira DM, Engelhardt E

Psychiatry and Neurology, Federal University of Rio de Janeiro, Rio de Janeiro, RJ, Brazil.

Vascular white matter lesions (WML) represent one of the main neuroimage findings in individuals older than 65 years and can lead to executive dysfunction and behavioral disorders.

***Methods:***Outpatients (n=20) with high severity WML evaluated with magnetic resonance imaging were selected using the Fazekas scale. Clinical data were described and correlated with demographic variables and ischemic score.

***Results:***Most patients (n=17; 85%) presented an altered Trail Making Test ratio (section B/section A); on Verbal Fluency, 15 individuals (75%) performed below the cutoff score. Apathy (5.9±4.65) and depression (3.05±3.67) were frequent as assessed by the Neuropsychiatric Inventory. The impairment in functional activities strongly correlated with apathy (r=0.814, p<0.001) and Verbal Fluency (r=0.744, p<0.001).

***Conclusion:***Executive dysfunction, apathy, and depression were the main characteristics found. Extension of WML may have distinct impact on the clinical picture, but further studies with methodological adjustments are necessary to provide more definitive conclusions.

## ABSTRACT – 5

**Changes of depressive symptomatology among nursing home patients - a 12 month follow-up**

Barca ML^1^, Selbæk G^1^, Engedal K^1^, Barca ML^2^, Laks J^2^

^1^Geriatric Department, Ullevål University Hospital, Oslo, Norway; ^2^Centre for Alzheimer’s Disease, Institute of Psychiatry - Federal University of Rio de Janeiro, Rio de Janeiro, RJ, Brazil.

Depression is prevalent among patients with dementia. Dementia progress over time, but our knowledge is limited regarding the course of depression. The aim of this study is to examine changes in depressive symptomatology among nursing home patients in a 12 months period.

***Methods:*** A sample of 901 nursing home patients was assessed with the Cornell Scale, the Clinical Dementia Rating Scale (CDR), a global scale for physical health and Lawton’s Scale for ADL. Information was collected regarding demographic characteristics and diagnosis. The patients were followed-up 12 months later. Multiple logistic regression was used to find predictors of death and multiple linear regression analysis to find predictors of worsening of total Cornell score.

***Results:*** During 12 month follow-up 233 patients died. Significant predictors of death were younger age (p<0.001), higher Cornell score (p=0.034), worse PADL score, (p<0.001), worse physical health (p=0.022) and suffering neoplasm (p=0.009). Of the 618 patients alive, 546 had complete data on Cornell Scale at baseline and follow-up. Mean Cornell score was 4.6 (SD 4.7) at baseline and 4.5 (SD 4.5) at follow-up. Compared to baseline 49.2% of the patients had a score higher or lower than 3 point at follow-up. 37.9% of the patients used antidepressants at baseline and 35% after 12 months; 30.6% had a persistent use (both at baseline and follow-up), 4.4% an incident use and 7.3% had the antidepressant withdrawn. If we consider a diagnosis of depression with Cornell score higher or equal to 8, the prevalence of depression at baseline and 12 months was both 21.2%, the persistence rate was 9.5% and the incidence and recovery rates were both 11.7%. The multiple linear regression analysis showed that shorter length of stay in nursing home (beta –0.08, p=0.028) and low Cornell sum score at baseline (beta –0.54, p<0.001) were associated with higher Cornell score after 12 months compared to baseline.

***Conclusion:***Higher score on Cornell Scale was associated with mortality after 12 months. Predictors of worsening of depression as measured by Cornell Scale were shorter stay in nursing home and lower Cornell score at baseline.

## ABSTRACT – 6

**Psychoactive drugs acting on the major cytochrome P450 isoenzymes in the elderly**

Cabrera MA, Dip RM

Internal Medicine, Universidade Estadual de Londrina, Londrina, PR, Brazil.

The objective of this study was to analyze psychoactive drugs (PD) acting on the major cytochrome P450 (CYP450) isoenzymes that are used daily by non-institutionalized elderly individuals.

***Methods:*** This is a cross-sectional population-based study with elderly individuals (60 years old or more). All continuously used psychoactive medications (antidepressant, antipsychotic, anticonvulsive and sedative) with hepatic metabolism via CYP450 isoenzymes – CYP1A2, CYP2C9, CYP2C19, CYP2D6 and CYP3A4 that are classified as substrates, inducers or inhibitors were considered.

***Results:*** 396 elderly individuals (222 women; 174 men) between 60 and 95 years old (mean: 72.1) were studied. The use of psychoactive drugs was identified among 63 (15.9%). The action on different CYP450 isoenzymes was observed in according to two groups – psychoactive drugs (PD) and non-psychoactive drugs (NPD): CYP1A2 – 28.6% (PD) and 6.0% (NPD); CYP2C9 – 41.3% (PD) and 17.2% (NPD); CYP2C19 – 58.7% (PD) and 4.8% (NPD); CYP2D6 – 66.6% (PD) and 10.5% (NPD); CYP3A4 – 73.0% (PD) and 7.5% (NPD). The psychoactive drugs with action on isoenzymes were: CYP1A2 – Tricyclic and antipsychotic as substrate (SUB), fluvoxamine as inhibitors (INH) and phenobarbital an phenytoin as inducers (IND); CYP2C9 – phenyton (SUB), fluoxetine (INH) and phenobarbital (IND); CYP2C19 – tricyclic and benzodiazepines (SUB) and fluvoxamine (INH); CYP2D6 – tricyclic, fluoxetine, paroxetine, fluvoxamine, venlafaxine and antipsychotics (SUB) and fluoxetine and paroxetine as INH; CYP3A4 – tricyclic, antipsychotic, benzodiazepines and carbamazepine (SUB) and phenobarbital, phenytoin and carbamazepine as IND.

***Conclusions:*** The results showed that there was a high use of PD acting on CYP450 enzymatic system, thus increasing the risk of drug interaction in a group that is already vulnerable to adverse effects from drugs due to polypharmacy, aged chances and comorbidities.

## ABSTRACT – 7

Dimensions underlying the Mini-Mental State Examination in a sample with low education levels - The Bambui Health and Aging Study (BHAS)

Castro-Costa E^1,2^, Fuzikawa C^1^, Uchoa E^1^, Firmo J^1^, Lima-Costa M^1^, Ferri CR^2^

^1^Laboratorio de Epidemiologia e Antropologia Medica, Centro de Pesquisa Rene Rachou, Belo Horizonte, MG, Brazil; ^2^Stewart, Section of Epidemiology, King’s College London, London, United Kingdom.

To investigate the validity of previously suggested dimensions underlying the MMSE and differences in associations of these dimensions with socio-demographic and health characteristics in an older Latin American community sample with low levels of education.

***Design:*** Secondary analysis of baseline data from a population-based cohort study. *Setting:* Bambuí, Brazil. *Participants:* Of 1742 total residents aged 60 years or over, 1558 (89.4%) participated at this study. *Measurements:* A standard Brazilian version of the Mini-Mental State Examination (MMSE).

***Results:*** A five-factor solution (concentration, language/praxis, orientation, attention, memory) for the MMSE was generated from Principal Components Analysis and the five factor solutions proposed in previous studies of developed nation samples was supported in this sample by Confirmatory Factor Analysis. In the adjusted linear regression models, MMSE factors varied in their correlates: for example, female gender was associated with higher concentration, orientation and attention but lower language/praxis; increased age was only inversely associated with language and attention; ADL impairment was principally associated with lower language/praxis.

***Conclusion:***This study provides support for the cross-sectional equivalence of the MMSE, at least suggesting that most of the items and underlying constructs remain meaningful even after alteration and translation and lower overall distribution in a low education sample.

## ABSTRACT – 8

**A population-based study of the association between *Trypanosoma cruzi* infection and cognitive impairment in old age (The Bambuí study)**

Lima-Costa M^1^, Castro-Costa E^1,2^, Uchoa E^1^, Firmo J^1^, Ribeiro A^1^, Ferri C^2^, Prince M^2^

^1^Laboratorio de Epidemiologia e Antropologia Medica, Centro de Pesquisa Rene Rachou, Belo Horizonte, MG, Brazil; ^2^Section of Epidemiology, King’s College London, London, United Kingdom. Limited clinical data suggests that chronic *Trypanosoma cruzi* infection, which causes Chagas disease (ChD), is associated with cognitive impairment. This study investigated this association in a large population-based sample of older adults.

***Methods:*** Participants in this cross-sectional study comprised 1,449 persons aged>60 years from a Brazilian endemic area (Bambuí). Cognitive functioning was ascertained by the Mini-Mental State Examination (MMSE), considering its score in percentiles (<14 [<5th percentile], 15–22 [5^th^ to <25^th^] and>23). Hypothesized risk factors were *T. cruzi* infection, ChD-related electrocardiographic (ECG) abnormalities and use of digoxin medication. Potential confounders included depressive symptoms, smoking, stroke, haemoglobin, HDL cholesterol, blood glucose, systolic blood pressure, and use of psychoactive medication.

***Results:*** The prevalence of *T. cruzi* infection was 37.6%. There was a graded and independent association between infection and the MMSE score (Adjusted odds ratios estimated by ordinal logistic regression=1.99; 95% CI 1.43–2.76). No significant associations between the MMSE score and ECG abnormalities or digoxin medication use were found.

*** Conclusions:*** This study provides for the first time epidemiological evidence of an association between T. cruzi infection and cognitive impairment which was not mediated by either ChD-related ECG abnormalities or digoxin medication use.

## ABSTRACT – 9

**Linear and non-linear regional profiles of brain aging in non-demented elders: a voxel-based morphometric MRI study**

Curiati P^1^, Tamashiro JH^1^, Squarzoni P^1^, Duran FS^1^, Santos LC^1^, Vallada H^1^, Busatto GF^1^, Alves T^1^, Wajngarten M^2^, Leite CC^3^, Menezes PR^4^, Scazufca M^4^

^1^Psychiatry Department, Neuroimaging in Psychiatry Laboratory, Clinical Hospital, São Paulo University Medical School, São Paulo, SP, Brazil; ^2^Division of Cardiogeriatry, Heart Institute, Clinical Hospital, São Paulo University, São Paulo, SP, Brazil; ^3^Radiology Department, Clinical Hospital, São Paulo University Medical School, São Paulo, SP, Brazil; ^4^Epidemiology Section, University Hospital, São Paulo University, São Paulo, SP, Brazil.

Most cross-sectional morphometric MRI studies of brain aging have used linear methods to investigate relationships between age and regional GM indices. We aimed to ascertain whether age-related volumetric reductions occur in different degrees of severity and following different linear or non-linear models, what could partially explain the discrepancies in the results of previous investigations on normal aging.

***Methods:*** We used estimates derived from voxel-based morphometry region of interest masks to investigate the relationship between GM volumes (corrected for the total amount of GM in the brain) and age during elderly life (67–75 years) in 45 males and 57 females. Multivariate multiple regression analyses were performed to assess the goodness of fit of first, second and third order polynomial.

***Results:*** Our analysis revealed a linear pattern in the left amygdala, with males presenting accelerated GM decline and females presenting relative preservation. In the left occipital cortex, a linear regression model indicated relative preservation of this region in males. In the right occipital cortex a cubic regression indicated an increase in the regional vulnerability to aging in the eightieth decade. Regarding the parahippocampal findings, males presented an acceleration of GM loss in the left side and a steady decrease at the right side.

***Conclusion:*** Brain aging follows a gender-specific pattern and is heterogeneous in elderly individuals, with some regions presenting non-linear volumetric changes and others a more steady profile. The mapping of such variability provides a framework that may improve our understanding about structural brain abnormalities and allow the development of early diagnostic markers.

## ABSTRACT – 10

**MRI-based partial volume correction of positron emission tomography images in non-demented elders: accounting for the effects of brain atrophy when studying metabolic profiles**

Curiati P, Duran FS, Tamashiro JH, Squarzoni P, Buchpiguel CS, Santos LC, Wajngarten M, Leite CC, Vallada H, Menezes PR, Scazufca M, Busatto GF, Alves T

São Paulo University Medical School, São Paulo, SP, Brazil.

Despite technological advances, apparent radiotracer concentration in positron emission tomography (PET) images is influenced by regional gray matter concentration, a phenomenon known as partial-volume effect (PVE). We aimed to investigate the influence of atrophy correction on the profile of funcional brain aging in elders.

***Methods:***Voxel-based analysis of cerebral glucose metabolism (CMRglc) was performed in a sample of 30 elders (67–75 years), all classified according to ICD-10 as psychiatric and neurologicaly normal. The PVElab software was used to perform the PVE correction by co-registering magnetic ressonance and PET images. Correlations between age and 18FDG uptake were assessed and results before and after the correction were compared.

***Results:***Before PVE correction, there were significant negative correlations between age and CMRglc in the right cerebellum, left hippocampus/ parahippocampal region and right temporal cortex in males, whereas several prefrontal regions where involved in females. After correction, hippocampal and parahippocampal CMRglc changes in males did not sustain statistical significance, suggesting a major role of brain atrophy for limbic metabolic variability in elderly males. Conversely, negative correlations in these two specific structures emerged as a significant pattern of females only after PVE correction, suggesting that regional hypometabolism exceeds brain atrophy elderly females. Also, the maintenance of the other abovementioned findings provides support for the notion that regional CMRglc that is not completely secondary to volumetric changes in healthy elders.

***Conclusion:*** With the use of PVE correction, it is possible to differentiate metabolic changes that are secondary to brain atrophy from the ones that are intrinsic of the remaining cerebral parenchyma. The mapping of such metabolic profiles is crucial to a better understanding of degenerative processes that affect the brain and provides a framework that may improve our understanding about the brain abnormalities and allow the development of early diagnostic markers.

## ABSTRACT – 11

**The effect of exercise as an adjunctive treatment for depressed elderly: a 1-year follow-up study**

Deslandes A^1^, Coutinho E^1^, Deslandes A^2^, Moraes H^2^, Silveira H^2^, Mouta R^2^, Piedade R^2^, Ribeiro P^2^, Laks J^2^, Pompeu F^3^

^1^ENSP, FIOCRUZ, Rio de Janeiro, RJ, Brazil; ^2^IPUB, UFRJ, Rio de Janeiro, RJ, Brazil; ^3^EEFD, UFRJ, Rio de Janeiro, RJ, Brazil.

The effects of physical exercise on the treatment of depressive elderly adults have not been assessed by changes in cortical hemispheric activity so far. The present study aimed at evaluating changes in depressive symptoms, quality of life, and cortical asymmetry produced by aerobic activity. Twenty patients diagnosed with Major Depressive Disorder (MDD) were divided into a control group (undergoing pharmacological treatment) and an exercise group (pharmacological treatment plus aerobic training) in a quasi-experimental design. Subjects were evaluated by depression scales (Beck, HAM, MADRS), SF36, and electroencephalographic measures (frontal and parietal alpha asymmetry), before and after 1 year of treatment. After one year, the control group showed a decrease in cortical activity on the right hemisphere (increase of alpha power), which was not observed in the exercise group. In relation to the depressive symptoms, the exercise group showed a significant decrease of depressive symptoms, which was not observed in the control group. This result was also seen by improved treatment response and remission rate after one year of aerobic exercise associated with the treatment. This study presented preliminary support for an effect of aerobic training on cortical activity and on depressive symptoms in elderly patients. Exercise facilitates the treatment of depressive elderly adults once it acts in the clinical and physical improvement of these patients and protects against a decrease in cortical activity.

## ABSTRACT – 12

Emotional responses to awareness of deficits in Alzheimer’s disease

Dourado M^1^, Engelhardt E^1^, Laks J^1^, McCallum T^2^

^1^Institute of Psychiatry of the Federal University of Rio de Janeiro, RJ, Brazil; ^2^Department of Psychology, Case Western Reserve University, Cleveland, OH, USA.

This study aimed to assess the emotional responses in patients with Alzheimer’s disease (AD) according to their awareness of cognitive deficits.

***Methods:*** The mild to moderate patients and their caregivers (n=52) were evaluated by a questionnaire-based method for deficit awareness and for the presence of its emotional response. MMSE, CDR, and Cornell for cognitive status, severity of dementia and depression, respectively.

***Results:*** The mild AD patients (52.2%) showed a more preserved awareness of cognitive deficits than the moderate group (17.2%). Most of the moderate patients (82.7%) do not recognize the impact of the symptoms. However, the assessment showed that 65.3% of the total dyads were aware of their emotional responses to the cognitive impairment and life changes. When compared by severity of disease there was no difference between CDR groups (p=0.47). The most frequent explanation they presented was that anger, sadness, and/or anxiety were related to the current inability to say or do things correctly.

***Conclusions:*** Mild and moderate patients presented emotional responses to the perception of memory decline and changes in life. Anxiety, sadness, and irritation were recognized as such in this group of patients, although awareness of deficits was more prevalent in the mild group.

## ABSTRACT – 13

**Comparison of Parkinson Disease Dementia prevalence rates according to diagnostic levels proposed by Movement Disorder Society**

Brucki SMD, Baldivia B, Batistela S, Esper JC, Duarte CA, Rocha MG

Neurology, Hospital Santa Marcelina, São Paulo, SP, Brazil.

Movement Disorder Society (MDS) developed clinical diagnostic procedures for Parkinson Disease Dementia (PDD), establishing diagnosis on two levels process. Level I consists in a brief evaluation conduced by a clinician, while Level II consists in neuropsychological evaluation which is more suitable to specify the severity of dementia. Although Level I could be considerated a brief and easy tool for DPD diagnosis, his sensitivity is not known.

***Methods:*** Ninety DP patients were submitted to diagnostic procedures proposed by MSD for PDD. At level I, cognitive functioning were measured by performance on lexical fluency tests, subtests of MMSE (serial 7s, drawing of pentagons and 3 word recall) and activities for dailing living was evaluated using Pill questionnaire. Level II was composed by comprehensive neuropsychological evaluation that included memory, executive functions and attention tasks. Functional activities were evaluated by Disability Assessment for Dementia (DAD) scale. MMSE and Beck Depression Scale (BDI) were used as screening tools on both of the procedures.

***Results:*** The study group consisted of 55 women and 45 men with a mean age of 67.4±11.22 years, mean age at on onset of disease of 57.22±17.7 years, mean years with PD of 7±5.48 years and mean years of schooling was 4.3±3.57 years. MMSE mean score was 23±4.19, BDI and DAD means score were 12.25±SD 8.7 and 71±7.18, respectively. The prevalence for PDD varied according to diagnosis procedures adopted. Using level I procedures, 5% of patients evaluated were PDD while Level II diagnosed 30% of the sample as having PDD.

***Discussion:*** The rates of PDD diagnosis were different according to process levels adopted: suggesting that the diagnosis of DPD is very low when the Level I is used as diagnostic criteria. It could means that Pill questionnaire and cognitive assessment by MMSE subtests and others screening tests are not sensible enough to do PDD. Moreover, it shows the importance to evaluate basic and instrumental aspects of daily living activities, educational and cultural aspects must be considered to cognitive domains functioning evaluation.

## ABSTRACT – 14

**Prevalence of Parkinson Disease Dementia according to clinical diagnostic criteria proposed by movement disorder**

Brucki SMD^1,2^, Baldivia B^1,2^, Batistela S^1,2^, Augusto CD^1^, Esper JC^1^, Rocha MG^1^

^1^Neurology, Hospital Santa Marcelina, São Paulo, SP, Brazil; ^2^Psychobiology, Universidade Federal de São Paulo, São Paulo, SP, Brazil.

Dementia associated to Parkinson’s disease prevalence (PDD) is aproximately 30% and annual conversion rate is about 15%.

***Objective:*** To describe the prevalence of PDD in Brazilian sample of PD patients.

***Methods:*** Ninety PD patients were submitted to neurological evaluation for assess clinical aspects of disease by using UPDRS, Hoehn & Yahr and Schwab & England scales. All patients also were submitted to comprehensive neuropsychological evaluation that included memory, executive functions and attention tasks. Functional activities were evaluated by Disability Assessment for Dementia (DAD) scale. MMSE and Beck Depression Scale (BDI) were used as screening tools. ANOVA compared performance of PD and PDD groups.

***Results:*** The study group consisted of 55 women, with mean age 67.4±11.22 years, mean age at on onset of disease of 57.22±17.7 years, mean years with PD of 7±5.48 years and mean years of schooling was 4.3±3.57 years. MMSE mean score was 23±4.19, BDI means score were 12.25±8.7. For Hoehn & Yahr, mean score was 3.5±7.6, for Swab & England 74.4±19.2 score and to UPDRS 50.8±21. The prevalence for PD-D found was about 30.2% for probable PD-D and 29.2% for possible PDD. ANOVA analysis compared performance of PD × PDD groups, revealing group effect for age (p<0.003; DPD>DP 64.5±10 years), age on onset of PD (p<0.03; DPD>DP), years of schooling (p<0.003; DPD DPD). There were no group effects for time of PD (p=0.32), severity of disease (p=0.09); DP and score mean on UPDRS (p=0.52). The prevalence of PDD found on sample was similar to whose reported by others studies. Age and age at onset of disease was identified as risk factors for PDD developing while high mean scores on severity of disease scales did not influenced on PDD diagnosis. It could be interpreted in function of high clinical impairments of disease on both of the groups. On the other hand, years of schooling seemed to be a protective effect on PDD incidence, reinforcing cognitive reserve concept. These results provide information about the contribution clinical variables on PDD prevalence.

## ABSTRACT – 15

**Comorbidity of elderly inpatient care**

Fernandes LS^1,2^, Palhinhas J^1^, Pinto A^1,2^, Ferreira J^1^, Lourenço H^1^, Fontoura M^1^, Ponte C1, Torres R^1^, Martins S^2^

^1^Psychiatry, Hospital of S. João of Porto, Porto, Portugal; ^2^UNIFAI, University of Porto, Porto, Portugal.

The elderly patients with multiple chronic diseases have an increased risk of having a worse quality of health care, when compared with of no comorbidity patients. For understanding this fact, is very important the difficulties of treatment for these multiple conditions (namely adverse effects), too many different recommendations or even guidelines overlapping for each individual pathology as well as the rising of expenses. When there is comorbidity, above all in addition to dementia, this leads to a great risk of death, overloading the health care, functional decline and worsening of the quality of life.

***Objective:*** Analysis of comorbidity of elderly inpatient care in Gerontopsychiatry of Hospital S. João of Porto, in Portugal.

***Methods:*** A cross-sectional assessment during two years was carried out, with socio-demographic characteristics, psychiatric pathologies, comorbidity and treatment of old patients in this hospital unit.

***Results:*** In a sample of 158 old patients, the most found psychiatric diagnosis were: dementia, bipolar disorder, depression, psychosis, alcohol addiction, delirium and anxiety disorders. The pharmacotherapy most used was the anxiolitics minor and major, and 81% of these patients had three or more medications simultaneously prescribed. There were comorbidity in 77.8% of these patients being cardiovascular, infectious and diabetes the pathologies most frequent. In most of these cases, one or more comorbidities with more use of anti-hypertension and anti-dislipidemic medication were associated and in 54.8% of the cases, there was one or more medications prescribed.

***Conclusion:*** In this inpatient sample of Psychiatric Service of General Hospital, medical and psychiatric characterization and the associated medication were in accordance to the majority of the studies in this field.

## ABSTRACT – 16

**Effects of attention, memory, and executive functions training on the quality of life and well-being of healthy elders**

Irigaray TQ, Schneider RH, Gomes I

Institute of Geriatrics and Gerontology (IGG), Pontifical Catholic University of Rio Grande do Sul (PUCRS), Porto Alegre, RS, Brazil.

***Introduction:*** The cognitive functioning of elders is related to their health, quality of life and well-being and is considered an important indication of active aging and longevity.

***Objective:*** To verify the effects of an attention, memory, and executive functions training program on the quality of life and psychological well-being of elders.

***Methods:*** 76 healthy elders participated in the study. The experimental group (EG) and the control group (CG) had both 38 participants. The EG received 12 training sessions in attention, memory and executive functions. Training involved information on attention, memory, and executive functions and aging, as well as exercise instruction and practice. Elders were individually assessed in pre- and post-test. They answered to sociodemographic questions and to questions on cognitive functions (Mini-Mental State Examination – MMSE, NEUPSILIN Brief Neuropsychological Assessment Instrument, and Wisconsin Card Sorting Test – WCST), on depressive symptoms (Geriatric Depression Scale – GDS-15), on anxiety symptoms (Beck Anxiety Inventory – BAI), on quality of life perception (WHOQOL-Bref), and on psychological well-being (Personal Development Scale – EDEP).

***Results:*** Using t-test for paired samples we found statistically significant differences between groups in pre- and post-test in the variables cognitive functions, quality of life and psychological well-being. Elders from EG presented better post-test performance in the following NEUPSILIN’s subtests: attention, memory, language, praxias, problem solving, and executive functions. This better post-test performance from EG elders was also observed in WCST, which is a measure of executive functions. In post-test the EG presented less anxiety symptoms and better perception of quality of life in the physical and psychological domains. As to psychological well-being the EG presented significant post-test improvements in EDEP’s environment, personal growing, self acceptation and generativity (generation) domains.

***Conclusion:*** We conclude that cognitive interventions can contribute to the improvement of quality of life and psychological well-being of elders.

## ABSTRACT – 17

**Assessing Qol in the elderly: The WHOQOL-OLD approach**

Irigaray TQ^1,2^, Trentini CM^2^

^1^PUCRS, Viamão, RS, Brazil; ^2^Federal University of Rio Grande do Sul, Porto Alegre, RS, Brazil.

The assessment of quality of life has received attention from different areas of knowledge within the last years. However, there is still no consensus on this construct. Specifically in relation to the quality of life in the elderly we verify a lack of specific measuring instruments. This idiosyncratic population presents singular aspects in its conceptualization of quality of life and in the determination of the conditions which contribute to its composition. The aim of this work is to present and describe the WHOQOL-OLD, which is a World Health Organization module to measure the quality of life in the elderly. The WHOQOL-OLD is a 24-item Likert scale instrument divided into 6 domains: 1) sensory abilities, 2) autonomy, 3) past-present-future activities, 4) social participation, 5) death and dying, and 6) intimacy. Each domain contains 4 items, which generate scores from 4 to 20 points. The 6-domain scores combined with the answers of the 24 items generate an overall score to the quality of life in elders. The instrument can be self-administered, interviewer administered or interviewer assisted. The WHOQOL-OLD must be administered as an additional module to the WHOQOL-100 or to the WHOQOL-BREF. It can be used in different kinds of research, epidemiological investigations, clinical trials, conduct efficacy studies, and in the implementation of services for the elderly. In conclusion, WHOQOL-OLD is an useful alternative with good psychometric properties to the investigation of quality of life in the elderly. Also, it comprises relevant aspects to assess the quality of life of the elderly which have not been taken into account by other instruments.

## ABSTRACT – 18

**Frequency of adverse drug reactions after a fast dosing regimen in antidementic treatment with memantine in a hospital setting**

Koeppel C, Stelzl C

Geriatrics, Wenckebach Klinikum, Berlin, Germany.

A slow increase in dose is recommended for initiation of antidementic treatment with antidementic drugs like memantine. If antidementic therapy is started during stay in a geriatric clinic with an average duration of 18 days a faster increase in dosing may be justified since the patient may be closely monitored for side effects by the geriatric team. We therefore studied the frequency of side effects in patients on a geriatric ward after a fast dosing regimen of memantine.

***Methods:*** 20 patients (mean age 83.4±2.4 years; 13 females, 7 males) admitted for delirium and suspected dementia were included in the study. Besides standard diagnostic procedures including a multidimensional geriatric assessment all patients were interviewed by a neuropsychologist and had a cerebral CT scan. All patients had moderate dementia most likely of Alzheimer type (15 patients) or vascular dementia (5 patients). None of the patients had been on antidementics before. In the patients 5 mg memantine twice a day was started for 7 days and than increased to 10 mg twice a day. Side effect were monitored by nursing staff and during the daily visit of the physicians.

***Results:*** Known side effects of memantine like dizziness, head ache, vertigo, gait disturbances, behavioral abnormalities, nausea or vomiting, and arrhythmia were not observed. There were no effects on blood pressure or pulse on initiation of treatment or dose increase.

***Discussion:*** In the patients included in the study no side effects were observed under a fast dosing regimen with memantine and a close side effect monitoring. This approach may have some advantages over a slow dosing regimen in an ambulant setting and may lead to earlier treatment effects.

***Conclusion:*** A faster dosing regimen for memantine in a hospital setting seems not to be associated with increased side effects and may have some advantages over the recommended slow dosing approach. Further study of this approach may be warranted.

## ABSTRACT – 19

**Frequency of adverse drug reactions after a fast dosing regimen in antidementic treatment with galantamine in a hospital setting**

Koeppel C

Geriatrics, Wenckebach Klinikum, Berlin, Germany.

A slow increase in dose is recommended for initiation of antidementic treatment with antidementic drugs like galantamine. If antidementic therapy is started during stay in a geriatric clinic with an average duration of 18 days a faster increase in dosing may be justified since the patient may be closely monitored for side effects by the geriatric team. We therefore studied the frequency of side effects in patients on a geriatric ward after a fast dosing regimen of galantamine.

***Methods:*** 21 patients (mean age 83.4±2.7 years; 14 females, 7 males) with suspected dementia of Alzheimer type were included in the study. Besides standard diagnostic procedures including a multidimensional geriatric assessment all patients were interviewed by a neuropsychologist and had a cerebral CT scan. 13 patients had mild, 8 moderate dementia. Dementia was most likely of Alzheimer type. All patients had not been on antidementics before. In the patients 4 mg galantamine twice a day was started for 7 days and than increased to 8 mg. Adverse drug reactions were monitored by nursing staff and during the daily visit of the physicians.

***Results:*** In none of the patients a change in eating behavior was observed. Side effects of galantamine like dizziness, head ache, vertigo, gait disturbances, behavioral abnormalities, nausea or vomiting, and arrhythmia were not observed. There were no effects on blood pressure, ECG, or pulse after initiation of therapy or increase of galantamine dose.

***Discussion:*** In the patients included in the study no adverse drug reactions were observed under a fast dosing regimen with galantamine and a close side effect monitoring. This approach may have some advantages over a slow dosing regimen in an ambulant setting and might lead to earlier treatment effects.

***Conclusion:*** A faster dosing regimen for galantamine seems not to be associated with increased side effects and may have some advantages over the recommended slow dosing regimen.

## ABSTRACT – 20

**Gain in ADL competence in geriatric patients with neuropathic pain after treatment with a combination of analgesics including pregabalin**

Koeppel C

Geriatrics, Wenckebach Klinikum, Berlin, Germany.

Neuropathic pain may seriously impair quality of life and ADL competence if it cannot be treated sufficiently. We studied patients admitted to a geriatric clinic for chronic neuropathic pain after optimized analgesic therapy including pregabaline.

***Methods:*** 15 patients (mean age 81.1±3.0 years, 11 females, 4 males) admitted for chronic neuropathic pain were included in the study. Besides standard diagnostic procedures including a multidimensional geriatric assessment all patients were interviewed by a neuropsychologist. All patients had polyneuropathy of diabetic or other origin. Pain was assessed by the visual analogue scale before, during, and after an optimization process of analgesic treatment including metamizol, opiates, antidepressants and pregabalin at a dose as low as possible. All patients were given 300 mg pregabalin besides a co-medication. Visual analogue scale and side effect monitoring were included. None of the patients had been on pregabalin before.

***Results:*** The dose requirements for the different analgesic drugs differed considerably in the individual patients. Pain could be reduced from an average of 5.5±2.1 to 2.5±1.3. Barthel index increased from 45±7 to 62±8 points after the optimization process which took up to 2 weeks. All patients stated on discharge that their quality of life had increased substantially.

***Discussion:*** Quality of life and ADL competence could be considerably improved by optimization of analgesic therapy including low doses of analgesics of different classes. Optimization was a highly individual process. Pregabalin played a major role in this context.

***Conclusion:*** A careful optimization process in analgesic therapy including NSAID, opiates, antidepressants and pregabalin at low doses may be helpful for controlling neuropathic pain, improve ADL competence, and quality of life.

## ABSTRACT – 21

**Aging and depression in a changing society: what are the risk factors?**

Kola L, Gureje O

Department of Psychiatry, University of Ibadan, Ibadan, Nigeria.

The proportion of elderly persons is growing most rapidly in developing countries. These countries are also undergoing social changes with potential impact on the health of the elderly. Using a multi-stage stratified sampling of households, we studied a representative sample of 2152 elderly persons, aged 65 years and over, resident in the Yoruba-speaking community of Nigeria. Depression was assessed using the world mental health version of the Composite International Diagnostic Interview. Disability was rated using the Sheehan Disability Scale and quality of life with the world health organization quality of life scale. We found a prevalence rate of 7.8% for 12-month DSM-IV major depression. Rates were unrelated to sex or to poverty. The odds of having depression increased with urbanization in a dose-response manner: compared with rural dwellers, persons living in semi-urban areas had increased risk while those living in urban areas had the highest rate. The levels of disability and quality of life impairment increased linearly with depression symptom severity. Work-related and home activities were most adversely affected but social roles were relatively preserved. Depression is a common problem among elderly persons living in this Sub-Saharan African country. Urbanization seems to be a risk factor. Depression affects both role functioning and quality of life even though traditional social support may vitiate its effect on social functioning. The growing population of the elderly and on-going social changes may contribute to make depression a major public health problem in Africa.

## ABSTRACT – 22

**Clinical assessment of Frontotemporal Dementia and Alzheimer’s disease. Neuropsychological performance, executive ability, patterns of glucose metabolism on FDG-PET and retention of the amyloid-imaging positron emission tomography (PET) tracer, Pittsburgh compound-B (PIB)**

Lindau MP, Degerman Gunnarsson M, Frizell-Santillo A, Nordberg A, Basun H, Lannfelt L, Kilander L Department of Public Health, Geriatrics, Uppsala, Sweden.

***Aim:*** The purpose was to compare the relation between cognitive and executive ability, illness awareness, regional glucose metabolism and retention of Pittsburgh compound-B (PIB) at baseline in nine patients with Frontotemporal Dementia (FTD), and eleven patients with Alzheimer’s disease (AD), and at follow-up of the AD patients after one year.

***Methods:*** Cognitive functions were investigated with neuropsychological tests, insight by interviews. Maximum insight score was 18 points. Cerebral glucose metabolism was assessed with positron emission tomography with FDG and the retention of beta-amyloid with PET-PIB. All AD patients were on acetylcholinesterase inhibitors.

***Results:*** At baseline all FTD patients except one were PIB-negative. The majority of the AD patients were PIB-positive. Cognitive ability differed between FTD and AD wih respect to tempo, naming and episodic memory. Illness awareness was considerably lower in FTD (mean 7.3, SD 7.4) than in the AD group (mean 12.0, SD 6). At follow up of the AD group, the cognitive ability was practically unchanged in one patient, selectively ameliorated as well as deteriorated in two patients, selectively worsened in three patients and generally lowered in four patients (one patient did not complete the follow up study). Levels of glucose metabolism and PIB were constant over time.

***Conclusion:*** A preliminary observation is that whilst glucose and PIB levels are constant at follow up in AD, neuropsychological evaluation shows cognitive deterioration in several cases. A more careful analysis will penetrate this contradiction.

## ABSTRACT – 23

**Comparing the characteristics of demented and non demented fallers in an acute care hospital**

Mamun K, Chou PC

Department of Geriatric Medicine, Singapore General Hospital, Singapore.

Several risk factors may predict fall, however it is not known if they are applicable to demented fallers as well. Our goal was to compare the risk profile of fallers with and without dementia so that more targeted fall prevention measure for demented patients.

***Methods:*** In one year period, all patients age 65 years and above, who had a fall during their hospital stay, were included in the study. Case note review and brief assessment of the patient was done by a geriatric trained nurse clinician within 12 hours of the incidence. Data of demented and non-demented fallers were compared to identify unique fall risk factors for demented patients.

***Results:*** Total 298 elderly patients, age 65 and above, fell over one year study period in our hospital, 50 of them had a diagnosis of dementia and 248 had no known diagnosis of dementia. Majority of patients used no ambulatory aids (64% demented patients vs 77.4% non-demented patients). In demented patients most fall occurred at night shift (40% demented vs 23.8% non-demented, p<0.009, χ^2^ test) whereas most non-demented patients fell in the morning shift (38% demented vs 61.3% non-demented). Morse fall risk assessment was done for all patients at the time of admission and demented patients had higher score (48.6 vs 40.21, p<0.028, T Test). More demented patients were admitted to surgical specialties than the non demented ones (32% vs 14%, p<0.009, χ^2^). At the time of fall, demented patients were more likely to have delirium than the non demented ones (94% vs 27%, p<0.000, Fisher’s Exact Test). Demented patients were more likely to be incontinent of urine (30% vs 18.3%, p<0.05, Fisher’s Exact Test) and visually impaired (38% vs 18.1%, p<0.004, Fisher’s Exact Test). Antipsychotics which were used more in the demented patients (10% vs 2.8%, p<0.03, Fisher’s Exact Test).

***Conclusion:*** Demented fallers are more likely to be confused, admitted to surgical specialities, visually impaired and incontinent of urine. They are also more likely to fall at bedside, at night, while trying to get up from bed, and on antipsychotic medications. Targeted fall prevention program addressing the above risk factors may reduce number of falls of hospitalized patients with dementia.

## ABSTRACT – 24

The introduction of augmentative and alternative communication in aphasia therapy

Marcolino J^1^, Oliveira J^1^, Deliberato D^2^

^1^Fonoaudiologia, UNICENTRO, Irati, PR, Brazil; ^2^Educação Especial, UNESP, Marília, SP, Brazil.

The aphasia is a disturbance of language after brain injury, common in the elderly. The treatment of patients with severe aphasia is limited. Sometimes the absence of speech is an obstacle to diagnose the aphasia. The patient with severe aphasia can not speak because the inability of articulation, like in dysarthria and/or apraxia. The speech therapist can not say if the language is impaired. The augmentative and alternative communication has been an effective method in the rehabilitation of these patients. This study describes the introduction of the augmentative and alternative communication in the therapy of two aphasic old subjects. Data analysis was composed of two parts: (1) The introduction of the augmentative and alternative communication in dialogue and (2) The use of reading and writing associated with symbols. The augmentative and alternative communication CSA was a support for the oral, reading and writing of these patients.

## ABSTRACT – 25

**Relationship between cognition and frontal EEG slowing in depressed and healthy elderly**

Mouta RS^1^, Deslandes A^1^, Silveira H^1^, Moraes H^1^, Capitão C^1^, Laks J^1^, Deslandes A^2^, Ribeiro P^2^, Piedade R^2^

^1^Center for Alzheimer Disese and Related Disorders - Institute of Psychiatry, Federal University of Rio de Janeiro, Rio de Janeiro, RJ, Brazil; ^2^Brain Mapping and Sensorimotor Integration Lab - Institute of Psychiatry, Federal University of Rio de Janeiro, Rio de Janeiro, RJ, Brazil.

***Purpose:*** To assess cognitive and electroencephalographic (EEG) findings in Major Depressive Disorder (MDD) and to evaluate correlations between cognition and frontal EEG slowing.

***Methods:*** We assessed 16 MDD (DSM-IV) and 10 healthy elderly. Our neuropsychological battery was composed by: Mini-Mental State Exam, Rey Auditory Verbal Learning Test, Rey-Osterrieth Complex Figure (ROCF), Category Fluency, Similarities Subtest of WAIS-R, Trail Making Tests A & B (TMT A & B), Digit Span Subtest of WAIS-R, Digit Symbol Subtest of WAIS-R and Stroop Test. We used 20 electrodes according to the international system 10/20 for the EEG acquisition. EEG data was analyzed by the average relative power scores of each frequency band. We calculated EEG power ratio (PR=(delta+theta) / (alpha+beta)) for each recording.

***Results:*** Depressed elderly showed lower performance on TMT A & B, ROCF and digit symbol. Cognitive performance was also related to EEG power ratio in both groups. However, PR index revealed no significant difference between groups.

***Conclusions:*** We found significant correlations between EEG slowing and executive functions in both groups. However, there was no significant difference in PR index. This fact might constrain its role in MDD while previous studies have supported its potential utility on dementia.

## ABSTRACT – 26

**Measuring anxiety in later life with the Geriatric Anxiety Inventory**

Pachana NA^1^, Byrne GJ^2^

School of Psychology, Univeristy of Queensland, Brisbane, Queensland, Australia; School of Medicine, University of Queensland, Brisbane, Queensland, Australia.

Anxiety symptoms and disorders are highly prevalent among older people, including those with Mild Cognitive Impairment. Our 20 item Geriatric Anxiety Inventory is a brief measure of dimensional anxiety specifically designed for use with older adults. The scale has demonstrated good reliability in both normal community and psychiatric samples (Cronbach’s alpha 0.91 and 0.93, respectively). Inter-rater and test-retest reliability are excellent, and the measure is well tolerated by older adults across a range of settings including residential care. Receiver operating characteristic (ROC) analysis indicated an optimum cut point of 8/9 to identify older patients with any anxiety disorder, correctly classifying 78% of patients (sensitivity 73%, specificity 80%). The GAI is in use in over a dozen countries with several translations in use, including Portuguese. A number of follow-up studies have indicated good pre-post utility in treatment studies on clinical populations. Uses and data for the current self-report scale in older patients with anxiety symptoms, and an introduction to an informant version in pilot testing phase, will be presented.

## ABSTRACT – 27

**Internet counselling for family caregivers of people with dementia: ‘Mastery over dementia’**

Pot A^1,2^, Willemse B^2^

^1^Netherlands Institute of Mental health and Addiction, Utrecht, Netherlands; ^2^VU University, Amsterdam, Netherlands.

The problems of family caregivers of people with dementia have been studied extensively. The results show a negative impact on mental health, varying from feelings of burden to psychiatric disorders including depression. Results on the treatment of caregivers’ psychological problems are less clear-cut. They show no or only modest benefits. Web-based interventions offer a very promising source of support, especially in view of the characteristics of the competing demands and limited time available to many caregivers. In addition, web-based interventions might be less stigmatizing compared to care provided by a mental health institute, which may be important for caregivers who often don’t see themselves as the ones who need help, because they are focused on the needs of the person with dementia. This presentation will be focused on an innovative web-based intervention, i. e. ‘Mastery over dementia’, for family caregivers of people with dementia. ‘mastery over dementia’ is a preventive intervention under the guidance of a professional counselor, and consists of 8 sessions and a booster session after one month. ‘Mastery over dementia’ is developed by the Netherlands Institute of Mental health and Addiction in collaboration with Alzheimer Netherlands and health care provider Foundation Geriant.

## ABSTRACT – 28

**Age and educational effects on category animal performance of normal elderly**

Fernandes CS^1^, Charchat Fichman H2, Nitrini R3, Caramelli P4, Lourenço RA5, Paradella E1, Carthery-Goulart MT3

^1^Universidade do Estado do Rio de janeiro, Rio de Janeiro, RJ, Brazil. ^2^Departamento de Psicologia, Pontíficia Universidade Católica, Rio de Janeiro, RJ, Brazil. ^3^Departamento de Neurologia, Universidade de São Paulo, São Paulo, SP, Brazil. ^4^Departamento de Neurologia, Universidade Federal de Minas Gerais, Belo Horizonte, MG, Brazil. ^5^Departamento de Medicina Interna, Faculdade de Ciências Médicas, UERJ, Rio de Janeiro, RJ, Brazil.

In Brazil, elderly population has grown significantly in the last decade. Normal aging process can lead to cognitive decline, including impairment of executive functions. In addition to the fact that Brazil is a country with increased life expectancy, a marked heterogeneity of educational level. Category Animal Fluency (CAF) is a frequently used test to detect cognitive impairment, assessing executive function, (mental organization, strategies for search), semantic and working memory, speed processing, language (size of vocabulary) and semantic memory and also can affect by education besides their clear influence on general cognition.

***Objective:*** To investigate the effect of age and educational level on Category Animals Fluency tasks (CAF) in healthy elderly and to discuss the different systems and cognitive functions involved in normal aging.

***Methods:*** The sample consisted of 319 healthy elderly, divided into two categories of age and five of schooling, which were evaluated at the outpatient care units of two university reference centers of Rio de Janeiro and São Paulo. To be included participants must have had preservation of global cognitive functioning, independence for the activities of daily living and had not fulfilled criteria of dementia. All participants were submitted to neurological and neuropsychogical evaluation.

***Results:*** There was a negative correlation between age and CAF performance (r= –0.26, p<0.01), which was not confirmed when years of education were included as covariant in univariate ANCOVA (F=0.50, p=0.48). Significant differences were found in CAF performance between educational level groups in correlation analysis (r=0.42, p<0.01) and ANCOVA analysis (F=12.1, p<0.05). Illiteracy was associated with the worst performance than any other schooling groups (p<0.01), while university level was associated with the best CAF performance when compared to other educational level groups (p<0.01).

***Conclusion:*** The most important improvement of CAF performance was found in two education stages: on the first years of schooling (literacy learning process) and when finishing high school and starting university courses. These stages are associated with the semantic memory and executive function improvement significant for Verbal Fluency performance. This observation points to the importance of literacy for cognitive development and to the conclusion of high school as a second marker for the refinement of cognitive abilities.

## ABSTRACT – 29

**Chronic stress is associated with elevated cortisol levels in Mild Cognitive Impairment**

Souza-Talarico JN^1,2^, Chaves EC^1^, Nitrini R^2^, Caramelli P^3^

**1**Medical-Surgical Nursing, School of Nursing, University of São Paulo, SP, Brazil. **2**Behavioral and Cognitive Neurology Unit, Departament of Neurology, Faculty of Medicine, University of São Paulo, SP, Brazil; **3**Behavioral and Cognitive Neurology Unit, Department of Internal Medicine, Faculty of Medicine, Federal University of Minas Gerais, Belo Horizonte, MG, Brazil.

High levels of cortisol, a stress hormone, in subjects with cognitive impairment have been reported as a consequence of a feedback inhibition lack in the hypothalamic-pituitary-adrenal (HPA) axis. However, considering that subjects with Mild Cognitive Impairment (MCI) may present chronic stress due to the awareness about their cognitive deficits, it raises the question whether, in these subjects, the elevated cortisol levels are also associated with daily chronic stress. This is particularly important since elevated levels of cortisol have been associated with memory deficits in the aging process. In this line of view, the current study aimed to verify the relationship between cortisol levels, chronic stress and coping strategies in MCI subjects. Basal morning salivary cortisol was measured in a sample composed of 41 healthy elderly and 33 individuals with amnestic MCI. Chronic stress was evaluated with the Stress Symptoms List (SSL) while the coping strategies were assessed using the Jalowiec Coping Scale (JCS). A two-way ANOVA revealed that those MCI subjects with high SSL scores presented higher cortisol levels (mean=312.9 ng/dl) compared with healthy elderly controls (mean=232.5 ng/dl; (F (1,70)=4.16; p=0.045). Moreover, those MCI subjects who elected emotion-focused coping showed higher SSL scores (mean=57.8) than those who elected problem-focused coping (mean=41.5; (F (1.66)=5.44; 0.023). In addition, a positive correlation between SSL scores and memory complaint even controlling for depressive symptoms was observed in the MCI (r=0.602) and control group (r=0.486, p<0.001). The association between chronic stress symptoms and high cortisol levels in the MCI group provide support that the daily stressful situations encounter by these individuals may contribute to increase the cortisol secretion. The current results also suggest that the chronic stress symptoms could vary as a function of the awareness about their cognitive deficits.

## ABSTRACT – 30

Proton spectroscopy and neuropsychiatric symptoms in Alzheimer’s disease and cognitive impairment non-dementia: a community-based study

Tascone LS^1^, Azevedo D^1^, Tatsch M^1^, Bottino C^1^, Castro CC^2^

^1^Psychiatry, FMUSP, São Paulo, SP, Brazil; ^2^Radiology, FMUSP, São Paulo, SP, Brazil.

***Background and Objective:*** The pathophysiology and the neurobiology of the neuropsychiatric symptoms in Alzheimer’s disease (AD) are far from understood. The aim of the study was to describe the findings of proton magnetic resonance spectroscopy (1H-MRS) in Alzheimer’s disease and cognitive impairment, non demented elderly (CIND) from a community-based sample.

***Methods:*** The dementia epidemiologic study was performed in the urban area of São Paulo. Thirteen patiens with AD, 11 with CIND and 13 normal individuals were evaluated. The 1H-MRS was performed in the right temporal, left parietal and medial occipital region studying the metabolits: N-acetylaspartate (NAA), creatine (Cr), choline (Cho) and myo-inositol (mI). The neuropsychiatric symptoms were assessed with the Neuropsychiatric Inventory (NPI) and the results correlated with the 1H-MRS metabolities.

***Results:*** Occiptal NAA, parietal Cr and occiptal mI were correlated negatively with NPI 10 in patients with AD (Spearman’s coefficient; p≥0.05). There was no correlation in CIND group. Occiptal Cho was correlated negatively with NPI 10 and 12 in control individuals (Spearman’s coefficient; p≥0.01).

***Conclusion:*** The results suggest that neuropsychiatric symptoms can be associated with especific metabolic alterations measured by 1H-MRS in patients with AD and normal elderly.

## ABSTRACT – 31

**Burden of care and emotional exhaustion mediate mental health status of Alzheimer’s disease caregivers**

Truzzi A, Souza W, Berger W, Bucasio E, Figueira I, Engelhardt E, Laks J

Center for Alzheimer’s Disease and Related Disorders, Instituto de Psiquiatria da UFRJ, Rio de Janeiro, RJ, Brazil.

Burnout is a syndrome in response to chronic interpersonal stressors at the work environment. AD caregivers face high levels of burden of care, which make them vulnerable to burnout. The aim of this study is to evaluate the prevalence of burnout and analyze its correlations to the clinical and sociodemographic characteristics of a sample of Brazilian AD caregivers.

***Methods:*** AD patients and caregivers (n=108) were consecutively included in our sample. Burnout and each dimension (emotional exhaustion, depersonalization and reduced personal accomplishment) were correlated to the caregivers’ sociodemographics, burden of care, anxious and depressive symptoms, and to the patients’ cognitive, functional, and behavioral profile. A regression model was applied using burnout, including each dimension independently, and burden of care as dependent variables.

***Results:*** Approximately five percent of caregivers experienced burnout. Burden of care was the only variable that showed the strongest association to caregiver burnout. Emotional exhaustion was the most prevalent dimension (43%) and correlated significantly to the burden of care (r=0.65, p<0.05), depressive (r=0.52, p<0.01) and anxious (r=0.52, p<0.01) symptoms in caregivers. The regression model showed that only burden of care explained the severity of burnout and its dimensions and emotional exhaustion explained the severity of caregiver burden.

***Discussion:*** Emotional exhaustion was the main feature of burnout in caregivers and was intimately associated to depression and anxiety in this group. Individual treatment strategies focusing on caregiver burden and emotional exhaustion may help reducing psychiatric morbidity and burnout in AD caregivers.

## ABSTRACT – 32

**Successful aging and related factors in a sample of urban-dwelling elderly Brazilians**

Chaves M^1^, Padua AC^2^, Eizirik C^2^, Kaye J^2^

^1^Hospital de Clínicas de Porto Alegre, Porto Alegre, RS, Brazil; ^2^Oregon Health & Science University, Portland, OR, USA.

The concept of successful aging is multidimensional, including the avoidance of disease and disability, the maintenance of high physical and cognitive function, and sustained engagement in social and productive activities. The Brazilian epidemiologic transition - rapid increase in life expectancy caused by the substitution of heart disease and cancer for infectious and parasitic diseases as causes of death- reflects several epidemiologic transitions that are intricately connected to socioeconomic and demographic differences. In this context, normal and successful aging are issues of great interest in helping us to understand how aging Brazilians adapt to these changes, and which health-related and socioeconomic policies can better serve elderly persons. The association of successful aging with demographic, socioeconomic, and medical characteristics in healthy, community-dwelling Brazilian individuals aged 60 years and older (N=345) was investigated. Participants were classified as successful (N=214, 62%) or normal agers (N=131, 38%). Successful agers participated in significantly more leisure activities (34%) than did normal agers (21%). Multivariate logistic regression analysis revealed that the fewer children living were a risk factor, while confidants and family income were protective factors for successful aging. This finding validates the concept that, in developing countries such as Brazil, higher income, a higher number of confidants, and fewer children living may prevail over biological determinants, such as age and parental longevity, to achieve successful aging.

## ABSTRACT – 33

**Patterns of dementia observed in a city mental hospital in India**

Acharya AK^1^

^1^Psychiatrist, Calcutta Pavlov Hospital, Kolkata, West Bengal, India.

Dementia cases are not infrequent in a busy hospital OPD in Calcutta. It accounts for 1–2% of total cases attending in a Calendar year in 2007–20008. Alzheimer’s disease is main cause in about 60% of cases, 20% belongs to the Vas. Dementia group, 15 mixed vas+ Alzheimer’s dementia, & DLB account for 5% of cases in both sexes, and 5% remaining cases are traumatic, inflammatory or HIV Dementia. Age variation is 58–78 years, with a median age 68 years, and mean age 66 years. There are 58% female & 42% male sufferers. There onset of illness & attending to Hospital OPD varies from 6 months to 3 years. Diagnosis is clinical in >50% cases. Imaging like CT SCAN of brain is done in 40% of case. 10% cases can undergo an MRI SCAN. Both facilities is lacking in the Hospital. They are brought by their spouse in 65% of cases followed by son, daughter, & grand sons, hardly by neighbors too.

***Methods:*** About 2240 new cases were registered in the OPD of this Hospital. 200 dementia cases from 1.12.07–12.12.07 were taken for studies,here. 120 Alzheimer’s with no hypertension or other brain pathology were noted in the first group. There were 62 female & 58 males. The 11-item MMSE is administered by the psychologist & there score range were 11–23. Mild dementia accounts for >70% case. Moderate dementia with score below 15 were 20–22%. Severe dementia are rare to bring them to hospital OPD & They accounts for less than 8%. 38 vas. dementia were 26 male &12 female, & their age ranges are 52–70 years with mean of about 60 years. Of the other dementia 28 mixed dementia cases, m: ratio is almost same. There were 1 HIV+ dementia in late stage, diagnosed & later died, there were 4 DLB cases with florid Parkinsonism feature, fluctuating cognition & hallucinations, & 2 male also diagnosed clinically as DLB dementia. There were 3 traumatic dementia after road traffic accidents. One ex-soldier had a multiple bullet injury history. Dementia following viral fever could not be traced in this study. Management & care: 2 persons send by judiciary & police were treated in the hospital. Rest were managed at home, spouses accounts for >60% care, >20% son or daughter-in-law or daughter is involved. Nobody is kept in old age home.

***Conclusion:*** Patterns of dementia in an urban mental hospital opd coforms with other similar situations elsewhere.

## ABSTRACT – 34

**Better mental health care in depressed elderly by diagnosing & managing them early**

Acharya AK^1^

^1^Psychiatry, Calcutta Pavlov Hospital, Calcutta, West Bengal, India.

***Introduction:*** Depression is very common in elderly in both sexes. But it usually remains masked or mixed with several other co-morbid physical disorders, & their non improvement or deterioration may points towards depression. Early dementia amalgamated with depression may worsen the scenario. Providing better mental health care to these elderly groups remain a challenge, even today. It depends on 1) early detection, 2) early intervention, 3) biological as well as supportive psychotherapy & vocational care or rehabilitation, 4) some governmental/ social support system.

***Methods:*** 120 cases recorded in Calcutta Pavlov Hospital, those were diagnosed as having depressive disorder as per DSM-4 diagnostic criteria, & in the age group of 65–73 were critically reviewed as to their degree of mental health care in the aspects of 1) diagnosis & duration of suffering prior to, 2) initiation of care, 3) patterns of care they are receiving, 5) social support and their quality of life at home, 6) nutritional status, 7) their hope & sense of future. There were 68 male & 52 female in the age group 65–73 with median age group of 69 years.

***Results:*** Those diagnosed early with symptoms arisen between 4–6 months, prior, were hopeful & relieved much applying A. Montegers scale. Those diagnosed late & initiation of treatment delayed shows less improvement. Nutrition is poorer in both the groups in comparison to other healthy control of same age group. Care was mostly anti-depressants and mood stabilisers, with occasional sedatives like lorajepam at bed time. Psychotherapy was instituted in only <25% case with history of suicidal ideas. Support at home & community is fair. Quality of life is good in first group.

***Conclusion:*** Early screening, diagnosis and combined biological, psycho-social and ideal home and social support can give the better mental health care in elderly depressed. Awareness of possibility of depression can assure a better mental health care.

## ABSTRACT – 35

**Linguistic performance of patients with Alzheimer’s disease and of children with Specific Language Impairment: a comparative study**

Alegria RP^1^, Pechman P^2^, Marques RC^2^, Perroco TR^2^, Bottino CM^2^, Nogueira MI^1^

^1^Neuroscience and Behaviour, University of São Paulo, São Paulo, SP, Brazil. ^2^Psychiatry, University of São Paulo, São Paulo, SP, Brazil

Language impairment in Alzheimer’s disease has been extensively studied and it is important to compare with challenging studies such as Specific Language Impairment (SLI) – defined as a developmental disorder of language – can also help to contribute to the early antecedents of later life language declinium.

***Objective:*** To compare the linguistic performance of Alzheimer’s disease patients and of children with Specific Language Impairment.

***Methods:*** Research on databases such as Medline/PubMed and SciELO, reviews of the last five years using the terms Specific Language Impairment, linguistic performance were analyzed for the Specific Language Impairment study. Six patients with Alzheimer’s disease were from the Old Age Group Ambulatory Care of The Institute of Psychiatry, Hospital das Clínicas, University of São Paulo. They were men and women aged 80 and older, with Mini-Mental State Exam (MMSE) scores of 13 to 26. Their oral discourses were recorded for fifteen minutes. The frequency of grammatical classes as prepositions, pronouns and conjunctions were analysed in the transcriptions. The computational tool Stablex, based on the mathematical-statistical-computer-assisted program, which proves to be very important for the lexical, textual and discursive analyses, was used for the linguistic performance of the patients.

***Results:*** We have found that the children with Specific Language Impairment (SLI) have very low linguistic performances compared to the children with normal language development, especially when they use prepositions and conjunctions and the research also found out that these class of words are the last to be learned by children with normal language development. On the other hand, the statistical analysis by the Stablex, the frequency of the grammatical class as prepositions, pronouns and conjunctions were also very low, probably because these words do not have semantic characteristics and they have not specific meanings.

## ABSTRACT – 36

**Depression and cognitive decline in vascular disease: preliminary results**

Alves CO, Alves GS, Kenjo KS, Lanna M, Valente L, Engelhardt E, Laks J

Psychiatry, IPUB, Rio de Janeiro, RJ, Brazil.

Depression in the elderly people has been associated to functional and cognitive impairment. Recently researches have studied the impact of the mood disorder on cognition and activity of daily life in patients with cognitive decline and vascular disease.

***Objectives:*** To correlate the intensity of depression symptoms with cognitive and functional impairment in a group with vascular Mild Cognitive Impairment (vMCI) and mild/moderate Vascular Dementia (VaD).

***Methods:*** Outpatient sample with late-onset depression (n=38) was divided in two groups vMCI (n=16) and VaD (n=22), age ≥65 years, Hachinski ≥4, scholarship vMCI=5.6 SD 4.3 / VaD=5.5 SD 5.3. The depression diagnosis was made according to the DSM-IV, MCI in agreement with Petersen and VaD based on NINDS-AIREN criteria. The groups were similar in age, scholarship and intensity of depressive symptoms. The association between scores of depression and dementia stages was compared using the Hamilton Depression Scale (HAM-D) and the Cornell Scale for depression in dementia (Cornell), and Clinical Dementia Rating (CDR). Neuropsychological tests – MMSE, CAMCOG, TMT A e B, Porteus, CDT e semantic Verbal Fluency performed, and Pfeffer’s Functional Questionnaire. The brain white matter was assessed with Fazekas scale.

***Results:*** The scores of depression scales were associated with worst result of tests which evaluate the frontal executive function (Porteus) – (HAM p<0.04/S0.449) and Cornell (p<0.03/S0.410) in VaD group. The functional impairment was associated with depression in the sample with vMCI (HAM p<0.001/S0.626) and Cornell p<0.001/S0.640).

***Conclusions:*** These preliminary findings favor the hypothesis that depression might be associated with specific neuropsychological deficits and impairment activity of daily life in elderly patients with vascular disease and cognitive decline.

## ABSTRACT – 37

**Effects of motor intervention on postural control associated with cognitive tasks: preliminary results**

Andrade LP, Stella F, Coelho FG, Barbieri FA, Gobbi LT, Gobbi S

Universidade Estadual Paulista, Rio Claro, SP, Brazil.

Patients with Alzheimer’s disease tend to present lower performance on balance tests than people without dementia, mainly during the execution of concurrent tasks. Increased oscillation posture seems to be associated with increased risk of falls. Motor and cognitive interventions could improve oscillation in postural control. The objective of the study was to analyze the effects of a motor intervention consisting of physical and front cognitive activities in patients with Alzheimer’s. Postural control was evaluated in four patients (mean age: 79.1 years, the Mini-Mental State Examination: 18.57±5.1, Clinical Dementia Rating Scale: stage 1.5±0.5 points, the Clock Drawing Test: 5.5±3.1 points) before and after an intervention program with duration of four months. The assessment of cognitive and postural control was performed on different days. In the assessment of postural control patients remained in four conditions on a force platform: 1) gaze directed at a target at eye’s height and arms vertically extended along the body, 2) previous condition with concomitant cognitive task (countdown starting in 30s), 3) condition “1” and holding a tray, 4) condition “3” and with concurrent cognitive task. At this four conditions was analyzed the variable area of center of pressure (CoP) by the Wilcoxon test (p<0.05). The patients presented lower area of center of pressure for the condition 4 (z= –1.96, p<0.05) and trend of lower area to the condition 2 (z= –1.72, p<0.08) after the motor intervention. The motor intervention provided reduced of body oscillation when concurrent task was performed, that suggest the contribution of attenuation of the risks of falls.

## ABSTRACT – 38

**Psicoative drugs used by institucionalized elderly in Fortaleza - CE**

Silva LM, Rodrigues PR, Andrade AS, Teles FM, Vasconcelos AM, Aragão LP

Universidade de Fortaleza, Fortaleza, CE, Brazil.

Elderly have singularities about their co-morbidities and medications use. Ninety percent of them use at least one drug what provide more risk to adverse events. In addition, institucionalized elderly has particular characteristics because of high prevalence of neurological and psychiatric disturbances and, so, they took more psicoative substances than general population. Transversal study with social, economics and demographics variables and also a medication list scheduled to institucionalized elderly in a house called Lar Torres de Melo in Fortaleza-CE. Al drugs listed were classified in a general group of psicoative pharmacs. Residents of the Institution for Long-Permanency of elderly were characterized by: 51.8% are women, mean age of 74.6 years and 74.32% receive social benefits and their mean of residence years in the referred institution is 7.82. They use about 3.57 medications and 33.8% use five or more drugs. In the sphere of psicoative pharmacy, 59.9% of elderly are users and the mean of those types is 1.56. Major categories described in this study are: benzodiazepines, anti-convulsivants and anti-depressives. This population has particular profile because of high prevalence of dependency and multiples co-morbidities. The study confirmed the literature that provide evidence about use of pscioative drugs in elderly who lives on a specialized institution of long-permanency-care.

## ABSTRACT – 39

**Influence of education and depressive symptoms on cognitive function in the elderly**

Avila R^1^, Moscoso M A^1^, Ribeiz S^1^, Arrais J^3^, Jaluul O^2^, Bottino CM^1^

^1^Psychiatry, University of São Paulo, São Paulo, SP, Brazil. ^2^Internal Medicine, University of São Paulo, São Paulo, SP, Brazil. ^3^Mathematic and Statistic, University of São Paulo, São Paulo, SP, Brazil.

The purpose of the present study was to investigate the influence that education and depression have on the performance of elderly on neuropsychological tests.

***Methods:*** This study was conducted at the Institute of Psychiatry, University of São Paulo School of Medicine, Hospital das Clínicas. All of the individuals evaluated were aged 60 or older. The study sample consisted of 59 outpatients with depressive disorders and 51 healthy controls. We stratified the sample by level of education, defining a low level of education as having had one to four years of schooling and a high level of education as having had five or more years of schooling. Evaluations consisted of psychiatric assessment, cognitive assessment, laboratory tests and cerebral magnetic resonance imaging.

***Results:*** We found that level of education influenced all the measures of cognitive domains investigated (intellectual efficiency, processing speed, attention, executive function and memory) except the Digit span forward and Fuld Object Memory Evaluation – immediate and delayed recall, whereas depressive symptoms influenced some measures of memory, attention, executive function and processing speed. Although the combination of a low level of education and depression had a significant negative influence on Stroop Test part B, Trail Making Test part B and Logical Memory - immediate, we found no other significant effects of the interaction between level of education and depression.

***Conclusion:*** The results of this study underscore the importance of considering level of education in the analysis of cognitive performance in depressed elderly patients, as well as the relevance of developing new cognitive function tests in which level of education has less effect on the results.

## ABSTRACT – 40

**Reliability and validity of the Cornell Scale for depression in dementia**

Barca ML^1,2^, Laks J^2^, Selbæk G^1^, Engedal K^1^

^1^Norwegian Centre for Dementia Research, Ullevål University Hospital, Oslo, Norway. ^2^Center for Alzheimer’s Disease, Federal University of Rio de Janeiro, Rio de Janeiro, RJ, Brazil.

***Aim:*** Depression is common in patients with dementia and is difficult to diagnose among severely demented patients, due to memory and language impairments. The most used scale to evaluate depression in these patients is the Cornell Scale. When it comes to validity and a cut-off for a diagnosis of depression different cut-offs are recommended, probably due to results from studies in different patient populations and different countries. The aim of this study is to determine the best cut-off of the Cornell Scale.

***Methods:*** Reliability study: The inter-rater reliability study will be carried out with 60 patients aged 65 years and over, both with and without dementia and recruited from two nursing homes (30) and a department of old age psychiatry (30) in two ways. Firstly, 30 patients will be assessed independently by two nurses that are primary carers of the patients within one week. In a second study one nurse will interview the primary carers of all 60 patients. In this session another nurse will be present. Both will rate the answers from the primary nurse with regard to the items of the Cornell Scale. The patients’ age, gender and degree of dementia according to Clinical Dementia Rating Scale (CDR) will be recorded.

***Methods:*** Validity study: The validity study will be carried out among 240 patients with and without dementia over 65 years in two memory clinics (60), three departments of old age psychiatry (60), one department of geriatrics (60) and two nursing homes (60). To assess depressive symptoms using the Cornell Scale trained study nurses will interview the patients’ primary caregivers/carers. Within the same week of this assessment the patients will be interviewed by a psychiatrist who has no access to the Cornell Scale evaluation. If possible an interview using the Montgomery Aasberg depression rating scale will be performed. To diagnose depression three diagnostic criteria will be used: ICD-10, DSM-IV and the Provisional diagnostic criteria for depression in Alzheimer’s disease. The patients’ age, gender, quality of life, ADL status, CDR, MMSE, use of medications and information about psychiatric and somatic diseases will be recorded.

## ABSTRACT – 41

**Depressive symptoms and associated factors in elderly community subjects**

Barcelos R

Old Age Research Group/ Proter, Institute of Psychiatry, Faculty of Medicine, University of São Paulo São Paulo, SP, Brazil.

To determine the frequency of clinically significant depressive symptoms (CSDS) in a community sample of Brazilian elderly and to assess their relationship with sociodemographic factors, cognitive and functional impairment (CFI), and clinical diseases.

***Design:*** Cross-sectional study of a community-based sample of elderly subjects. *Setting:* City of São Paulo, State of São Paulo, Brazil. *Participants:* 1,563 elderly subjects aged 60 years or older. *Measurements:* A 10-item scale for screening of depressive symptoms in elderly people (D-10), the Mini-Mental State Examination (MMSE), the Fuld Object Memory Evaluation (FOME), the Informant Questionnaire on Cognitive Decline in the Elderly (IQCODE), the Bayer Activities of Daily Living Scale (B-ADL), a sociodemographic and clinical questionnaire.

***Results:*** The frequency of CSDS was 13.0%. Univariate analysis identified independent factors associated with these symptoms in our sample. Logistic regression analysis indicated that being female, brown-skinned, previously depressed, having CFI, using psychotropics, and not practicing physical exercise were related to CSDS. On the other hand, being older, clinically sick, employed, or married were not associated with CSDS.

***Conclusions:*** Consistent with previous reports, female gender, lack of physical activity and CFI were significantly associated with higher frequencies of CSDS. Further investigations are necessary to clarify the occurrence of depression and possible modifiable factors in developing countries such as Brazil.

## ABSTRACT – 42

**SAFADIE: safety of acamprosate for alcohol dependence in the elderly**

Birkmayer F^1^, Kushner R^1^

Psychiatry, University of New Mexico, Albuquerque, NM, USA.

Alcohol dependence (EtOH dep. ) is highly prevalent and results in significant morbidity, mortality and cost to society. Its prevalence is increasing among the elderly. Although a few medications for EtOH dep. are available, there is little data on the safety or efficacy of these in the elderly. Acamprosate (ACP) is one of 3 FDA-approved medications for EtOH dep. ACP is not metabolized by the liver and has a unique safety profile compared to naltrexone or disulfiram, both of which can cause liver damage. Because the incidence of liver disease is high among alcoholics and increases with age and years of drinking, ACP, not being metabolized by the liver nor causing liver damage, may be safer for elderly patients with EtOH dep. SAFADIE is an open-label study of 25 subjects, age 60 and older who received ACP for 90 days with a primary outcome of rates of side effects and a secondary outcome of efficacy. The following results are based on 19 subjects. The average age of subjects was 68.2 years (standard deviation 7.19 y, range 60–81 y). 74% of participants were men, consistent with the observed gender ratio in prevalence studies of elderly drinkers. Every subject received ACP 666 mg tid, but 4 subjects reduced their doses due to adverse events. The overall rate of adverse events was 79% vs. 61% in prior studies (χ^2^=2.56, p=0.11, nonsignificant). Three subjects dropped out of the study, 1 because of side effects and 2 because of continued drinking and noncompliance with medication. The most commonly observed adverse events were diarrhea (37% vs. 16% in prior studies, χ^2^=5.77 df=1, p=0.016), pain (26% vs. 3%, χ^2^=30.27, p<0.0001), numbness (11% vs. 2%, c2=6.81 p=0.0091), leg cramps (11% vs. <1%, χ^2^=16.01 p<0.0001) and anxiety (11% vs. 6%, χ^2^=0.745 p=0.39, nonsignificant). It is unclear whether the higher rates of certain adverse events represent increased susceptibility to medication side effects (e. g. diarrhea) or are due to higher baseline rates of such conditions in the elderly (e. g. pain). With regards to efficacy, the percentage days abstinent as determined by time-line follow back increased by 28.4% (95% CI 14.6–42.3%, p=0.0006) from baseline to the last visit (90 d). With regards to perceived benefit, 47% of subjects reported a definite benefit (decreased amount/frequency/craving, fewer drinking days), 18% reported some benefit (decreased craving) and 29% had no benefit (drinking at same level as before). Although this study is limited by its open-label design and small number of subjects, it suggests that the overall rate of adverse events may be higher and specific side effects may be more frequent in the elderly, but also that a significant number of subjects reported benefit, even without psychosocial counseling. To our knowledge this is the first clinical trial of a medication for EtOH dep focused on the elderly. Further studies on the safety and efficacy of ACP and other medications for EtOH dep. in the elderly are needed.

## ABSTRACT – 43

**Effects of Electroconvulsive Therapy (ECT) on cognition and functionality of elderly patients with mood disorders in a Geriatric psychiatry ward**

Dias LM, Bottino CM, Hiratsuka M, Sitta MC, Jacob W

Hospital das Clínicas São Paulo, São Paulo, SP, Brazil.

***Introduction:*** Mood disorders are common in elderly and related with functional impairment and cognitive decline. Eletroconvulsive therapy (ECT) is effective to treat the humor symptoms; but there is few data about its impact on functionality and cognition.

***Objective:*** To evaluate the impact of ECT on functionality and cognition of elderly patients with mood disorders in a Geriatric psychiatry ward.

***Methods:*** Prospective-descriptive study in a Geriatric psychiatry ward with non-demented elderly with bipolar disorder or major depression submitted to ECT treatment. The subjects were assessed with the Mini-Mental State Examination (MMSE), Verbal Fluency (VF) and Clock Drawing Test (CDT) for cognitive evaluation. The Index of Independence for Activities of Daily Living (Katz’s Index), the Physical Self-Manteinance Scale (Lawton’s Index), the Activities Daily Life International Scale (ADL-IS), the Hang Grip and Timed Get up and Go Test (TGUG) were applied for functional assessment. The tests were applied before the ECT sessions and repeated at the discharge. The psychiatric symptoms were evaluated with Hamilton Rating Scale for Depression (HAM-D).

***Results:*** Eight subjects with 71±10.8 years old were included. They received an average of 14 ECTs sessions. Seven improved the psychiatric symptoms (HAM-D- 27.5±5.5 × 5.5±4.0 p<0.001 CI95%=15.9–28.1). Comparing before and after ECT there was improvement on MMSE (17.0±4.4 × 21.1±4.3, p=0.046, CI95%= –8.16 to –0.09), on VF (5.5±2.8 words/minute × 8.6±2.7 words/minute, p=0.034 CI 95%= –5.93 to –0.32). There is no difference on the Clock Drawing Test. We observed improvement on Katz’s Index (4.50±0.75 × 5.75±0.70, p=0.005, CI95%= –1.99; –0.50) Lawton’s Index (9.5±0.92 × 13.87±2.74, p=0.002 e CI 95%= –6.51; –2.23), ADL-IS (8.78±0.83 × 5.01±1.70, p<0.001 CI 95%=2.78; 4.74) and TGUG with average difference of 17.25 seconds (35.38±8.63 × 18.13±4.02 p<0.001 IC de 95%=11.95; 22.55).

***Conclusion:*** ECT in elderly with mood disorders is effective and it seems to have a positive impact on cognitive and functionality used tests.

## ABSTRACT – 44

**Executive functions in normal aging: the effects on the activities of daily living**

Faria C^2^, Fichman-Charchat H^4^, Fernandes C S^1^, Brooking L T^4^, Lourenço R A^3^, Paradela E^2^

^1^Psicologia, UERJ, Rio de Janeiro, RJ, Brazil. ^2^Instituto de Medicina Social, UERJ, Rio de Janeiro, RJ, Brazil. ^3^Faculdades de Ciências Médicas, UERJ, Rio de Janeiro, RJ, Brazil. ^4^Psicologia, PUC, Rio de Janeiro, RJ, Brazil.

The growth of the world-wide population with 60 years and older has been stimulating the study in this area. Studies point the cognitive functions decline as one of the main characteristics of aging. In special, impairment of executive functions seems to be more related to the aging, including normal aged. This cognitive process are responsible for the organization and regulation of mental processes and voluntary action, involving planning, monitoring, behavioral initiative and his anatomical correlate has been associated to prefrontal areas. In addition, studies indicate a significant contribution of the executive functioning for the accomplishment of the instrumental daily activities, as to prepare meals, to take medicines, to pay counts or to organize and plan the routine and the commitments. The difficulty on carrying them out can be cause of dependence in greater or minor degree. This study aimed at investigating the relation between executive functions and activities of daily living in normal aging. The sample consisted of 60 healthy elderly, between 66 and 94 years old, which were evaluated in outpatient care in university reference center from Rio de Janeiro (Center of Elderly Persons' Care-CIPI-UNATI from the State University of Rio de Janeiro). All participants were submitted to physical and neurological examination and to global cognitive evaluation with the Mini-Mental State Examination (MMSE). The instruments used in research were: Dementia Rating Scale, Digits Forward and Backward, Spatial Span Forward and Backward, Phonemic Verbal Fluency (PVF), Categorical Verbal Fluency-Animal Naming, Supermarket Fluency Test, Lawton Instrumental Activities Of Daily Living (IADL) Scale and Katz Basic Activities of Daily Living (ADL) scale. A Pearson correlation was conducted. Results shown a positive correlation between IADL and Dementia Rating Scale-Initiation/Perseveration Subtest (r=0.37, p<0.01), and between IADL and Supermarket Fluency (r=0.32, p=0.02). The results reveal that tasks that require executive functions are related with instrumental activities of daily living performance, especially mental and behavior organization, regulation, flexibility and initiative abilities. Besides, suggesting the importance of executive functions to independence in activities of daily living and to quality of life improvement.

## ABSTRACT – 45

**DSM-IV-TR diagnostic criteria for delirium: how to interpret criterion A?**

Cerejeira J^1^, Batista P^1^, Nogueira V^1^, Firmino H^1^, Mukaetova-Ladinska EB^2^

^1^Psychiatry, Hospitais Universidade Coimbra, Coimbra, Portugal. ^2^Institute for Ageing and Health, Newcastle University, Newcastle upon Tyne, United Kingdom.

Delirium is a frequent complication in medical or surgical elderly patients and has been associated to cognitive impairment and functional deterioration at follow-up. There is an overall agreement that delirium is far more frequent than it is recognized by medical or nursing staff. An important progress in the clinical diagnosis of delirium was the development of widely accepted criteria (DSM and ICD) and standardized instruments in order to assess patients with delirium. However, the sensitivity and specificity of these criteria for delirium are greatly affected by their interpretation and operationalization. For example, it has been unclear whether clouding of consciousness and inattention are both required symptoms, whether it is sufficient that either clouding of consciousness or inattention are present or if inattention is the clinical correlate for the concept of clouding of consciousness.

***Methods:*** This study included consecutive patients, aged 60 years or older, undergoing elective total hip replacement surgery in the Orthopaedics Department of Coimbra University Hospitals from October 2008. Cognitive function was assessed preoperatively with Mini-Mental State Examination (MMSE) and patients were excluded if DSM-IV-TR criteria for delirium were fulfilled. Patient’s mental state was assessed daily by a psychiatrist during 3 consecutive days, including the day of surgery, using a standardized instrument which is based on DSM-III-R criteria (Confusion Assessment Method, CAM) and confirmed with DSM-IV-TR criteria. The sensitivity and specificity of CAM criteria were determined against DSM-IV-R criteria using two definitions of criterion A (clouding of consciousness and inattention, clouding of consciousness or inattention).

***Results:*** The sample consisted in 66 patients, 30 males (45,5%) and 36 females (54.5%). Mean age was 73.95±6.237 and average preoperative MMSE was 26.09±3.294. Half of the patients showed an acute change in their mental status following surgery. Most frequent postoperative symptoms were psychomotor retardation (43,9%), altered levels of consciousness (27.3%) and impaired attention (16.7%). According to CAM criteria, 10 patients scored positively for delirium. From these, 9 patients were confirmed to have delirium using the more restrictive interpretation of DSM-IV-TR criteria (requirement of both clouding of consciousness and inattention). When using the alternative definition of criterion A (clouding of consciousness or inattention), 20 patients were diagnosed with delirium. These included all patients positively screened with CAM and lethargic patients with psychomotor retardation.

***Conclusions:*** DSM-IV-TR criteria requiring either clouding of consciousness or inattention are the most inclusive and their use is more likely to minimize the false negatives, especially in hypoactive delirium.

## ABSTRACT – 46

**Effects of age and sex on progression from Mild Cognitive Impairment to clinical dementia in the Chinese community – community versus volunteer samples**

Chiu HF^1^, Leung G^2^, Tam C^2^, Lui V^2^, Lam L^1^

^1^Department of Psychiatry, The Chinese University of Hong Kong, Hong Kong, China. ^2^Department of Psychiatry, Hospital Authority, Hong Kong, China.

Mild Cognitive Impairment (MCI) is a recognized risk factor for dementia. Variations in conversion rate to dementia across different population samples called for the need for refinement of current diagnostic framework.

***Objectives:*** In this study, we compared the progression rates to dementia in non-demented subjects (normal cognition and MCI) from both volunteers and community random recruit samples. The significance of demographic characteristics and its association with global cognitive deterioration were evaluated.

***Methods:*** 1106 Chinese persons (aged 60 or over) were recruited as volunteers or were community recruit participants of a recent population survey. All subjects were not demented at the baseline. At baseline and follow up at 2 years, each participant was assessed with Clinical Dementia Rating (CDR), Chinese version of Mini-Mental State Examination, list learning delay recall and category Verbal Fluency Tests (CVFT). Global cognitive status at follow up was classified as normal cognition (NC), MCI and clinical dementia.

***Results:*** At follow up, 0 and 28 (13%) of the NC and MCI subjects in the community sample progressed to clinical dementia. For the volunteer sample, 8 (76%) and 25 (22%) of the NC and MCI subjects progressed to clinical dementia. There was a significantly higher rate of progression in the volunteer sample (Pearson χ^2^=14.4, p<0.001). If age were controlled for, sample source was not related to cognitive outcome at follow up (Logistic regression, p=n. s. ). The older age group (≥75 years) showed significantly higher rates of cognitive deterioration. Older age, lower baseline MMSE and CVFT were associated with conversion to clinical dementia (Cox regression survival analysis).

***Conclusions:*** Variations in dementia conversion rates in different MCI samples may be related to sociodemographic confounders. Age remains a very significant factor determining rates of cognitive deterioration. This not unexpected finding indicates a practical concern of the importance of age stratification in subject recruitment for prevention trials of cognitive disorders in late life.

## ABSTRACT – 47

**Presentation of depressive illness in elderly in a rural clinic in India**

Acharya AK

Psychiatry, Calcutta Pavlov Hospital, Calcutta, West Bengal, India.

Depressive illnesses are the most common form of mental disorder observed in a clinic 124 km away from city capital of Kolkata. They, in the elderly>65 years of age are usually not present with low mood or low spirit or the usual symptoms, but are presented with a cluster of somatic complaints, those are reproduced in a same manner, those are thought as cardinal symptoms in these region.

***Methods:*** In Midnapore “Mind Care” clinic as described 124 km away from city 100 such depressive disorders cases of both sex were analysed for their presenting symptoms & patterns of recovery. Their age range was 65–77 years, and there were 64 females and 36 male patients, with a median age of 71 & mean age of 68 years.

***Results:*** Their presenting symptoms were:1) Headache 92%; 2) Neck-pain 62%; 3) Sleep disturbance 88%; 4) Multiple aches & pains 64%; 5) Musculoskeletal pain in extremities 52%; 6) Pain in genital regions 32%; 7) Loss of appetite 36%; 8) Diminution of libido 24%; 9) Crying 18%; 10) Suicidal thoughts 12%; 11) Pain in micturation 08%; 12) Pain in rectal or anal region 4%; 13) Pruritis vulvae (F) 02%; 14) Loss of interest to usual activities 56%; 15) Lack of pleasure 60%; 16) Sensation of a lump in throat or difficulty in swallowing 12%; 17) Backache or low abdominal pain 16%; 18) Burning in head & rinsing cold water for relief 18%; 19) Reeling of head 06%; 20) Irritability 36%; 21) Constipation, acidity & dyspepsia 40%; 22) Lack of memory & concentration 32%; 23) Fearfulness & beliefs of harm by others 12%; 24) Fear of diseases 20%.

***Conclusions:*** Atypical & somatic pain all over the body were the commonest presenting symptoms in both male & female elderly. Not only low mood or low spirit, but somatic unexplained pain features characterize the depressive disorders, which were diagnosed as per ICD-10 criteria.

## ABSTRACT – 48

**Verbal cued recall is a good predictor of conversion to Alzheimer’s disease in Mild Cognitive Impairment**

Dierckx E^1^, Engelborghs S^2^, De Raedt R^5^, Van Buggenhout M^4^, De Deyn PP^3^, Ponjaert-Kristoffersen I^1^

^1^Developmental and Life Span Psychology, Vrije Universiteit Brussel, Brussels, Belgium. ^2^Department of Neurology and Memory Clinic, Middelheim General Hospital (ZNA), Antwerp, Belgium. ^3^Laboratory of Neurochemistry and Behaviour, Institute Born-Bunge, University of Antwerp, Antwerp, Belgium. ^4^Department of Nursing Sciences, Faculty of Medicine, University of Antwerp, Antwerp, Belgium. ^5^Department of Psychology, Ghent University, Ghent, Belgium.

With the ongoing development of new disease-modifying treatments for Alzheimer’s disease (AD), there is an increasing need for early diagnosis. This study was set up to investigate whether neuropsychological tests are able to predict conversion to AD among Mild Cognitive Impairment (MCI) patients.

***Methods:*** At baseline the cognitive part of the Cambridge Examination for Mental Disorders of the Elderly (CAMCOG), the Mini-Mental Status Examination (MMSE), the Geriatric Depression Scale (GDS), a Dutch variation of Rey’s Auditory Verbal Learning Test (10-RAVLT; a 10-word learning task), the Memory Impairment Screen plus (MISplus; a verbal cued recall task) and the Visual Association Test (VAT; a visual cued recall task) were administered to 40 patients of a memory clinic diagnosed with MCI (according to Petersen’s criteria). After approximately 3.5 years, a follow up diagnosis was established. Of those who were seen for follow up (n=27), 17 fulfilled (NINCDS-ADRDA) criteria of probable AD (i. e. converters), while 10 did not convert to dementia (i. e. nonconverters) (conversion rate=63%).

***Results:*** A binary logistic regression analysis showed that the MISplus contributed most to the prediction of conversion (Wald c^2^ (1)=3.81, p=.05 (CI95%:.023–1.009). With a cut-off of 4 out of 6, a sensitivity of 82.4%, a specificity of 100%, a positive predictive value of 100%, a negative predictive value of 76.9% and an overall diagnostic accuracy of 88.9% were obtained.

***Conclusion:*** This prospective, longitudinal study showed that a score of 0,1,2,3 or 4/6 on the MISplus, a delayed verbal cued recall test, may be a good indicator of conversion to AD among MCI-patients.

## ABSTRACT – 49

**Awareness of disease in dementia: patients’ perceptions**

Dourado M

**Institute of Psychiatry of the Federal University of Rio de Janeiro, Rio de Janeiro, RJ, Brazil.**

***Background:*** Impairment of deficit awareness is a clinically relevant feature of dementia affecting the maintenance of decision capacities, management and safety of patients with risk behaviors, and caregiver burden. This study assessed awareness of disease of patient/caregiver dyads and the relationship between unawareness on various domains and sociodemographic variables among elderly Brazilians with Alzheimer’s disease (AD).

***Methods:*** The dyads (n=52), stratified by clinical severity and age groups, responded to the Assessment Scale of Psychosocial Impact of the Diagnosis of Dementia (ASPIDD). Statistical tests were used to compare clinical and sociodemographic variables and to calculate differences in rates of discrepant responses among mild and moderate dyads and between age groups, rates of discrepant responses among the ASPIDD domains, and association between awareness and age/age at onset.

***Results:*** Awareness of deficits did not differ significantly among mild patients, whereas moderate patients showed impaired recognition on all domains. Older moderate dyads showed more discrepant responses, as compared to younger dyads at both severity stages. Mild patients could associate the disease with the cognitive deficits and recognized impairments on other domains. There was no significant relation of awareness with age at onset.

***Conclusion:*** Mild AD patients could associate the disease process with the presence of cognitive deficits, and also the changes in the emotional response with difficulties in social, family, and affective relations. Moderate AD patients were less aware of the symptoms and did not attribute them to the disease.

## ABSTRACT – 50

**Depression and cardiac disease: co-occurrence and associated factors**

Drucker C^1^, Makdisse M^2^, Nasri F^2^, Blay SL^1^

^1^Psychiatry, UNIFESP, São Paulo, SP, Brazil. ^2^Hospital Israelita Albert Einstein - HIAE, São Paulo, SP, Brazil.

To investigate if the presence of cardiac diseases and metabolic changes are associated with depression in the elderly.

***Methods:*** A total of 516 elderly participants were evaluated in the cross-sectional Epidemiological Study on the Elderly Jewish Population/EPI-J, using structured questionnaires, applied face-to-face by trained examiners. Depression was diagnosed when self-reported depression and the identification of cases by the Geriatric Depression Scale (GDS-15) were simultaneously present. Other variables examined were: socio-demographic, life habit (levels of physical activities), social context, health (self-assessed) and metabolic characteristics (dyslipidemia). This study defined risk factors for dyslipidemia by the presence of one or more of the following criteria: high cholesterol (≥200 mg/dL), high LDL-cholesterol (≥160 mg/dL), use of hypolipemiants, high triglycerides (≥150 mg/dL), low HDL-cholesterol (<50 mg/dL in women or <40 mg/dL in men). Cardiac disease was considered positive if any of the following self-reported problems were indicated: valvular heart disease with hemodynamic repercussions, arterial fibrillation, heart failure, use of pacemaker, coronary artery disease and peripheral vascular disease.

***Results:*** A total of 20.5% of the sample showed cardiac problems, 19.4% showed depression and 27.5% the co-occurrence of both conditions. Multivariate analysis indicated that depression is associated with the female sex (logistic regression; OR 2.47; 95% IC 1.40–4.34); unsatisfactory self-assessed health (OR 3.07; 95% IC 1.78–5.31); longer sitting time on weekends (OR 1.00; 95% IC 1.00–1.002); risk of dyslipidemia (OR 2.84; 95% IC 1.61–4.99); cardiac problems (OR 1.86; 95% IC 1.06–3.26).

***Conclusion:*** In this study, sedentary females, with unsatisfactory self-assessed health, dyslipidemia and cardiac diseases, showed greater risk of association with depression.

## ABSTRACT – 51

**The importance of axis II diagnoses in elderly patients with major mental health problems**

Evans S^1,2^

^1^Psychiatry, East London Foundation NHS Trust, London, United Kingdom. ^2^Queen Mary University of London, London, United Kingdom.

Mental health services for older people rely substantially on published evidence to gain or retain funding for services. The myth that personality disorder is rare in older patients has not yet been fully dispelled and yet patients with significant axis II co-morbidity continue to require treatment. Their specific needs may be complex and demand particular skills from clinicians. This simple cross-sectional study identifies patients within a service who are co-morbid for axis II, and examines how this affects care, admission and risk.

## ABSTRACT – 52

**Cognitive and functional evaluation of elderly living in a long term care institution in São Paulo, Brazil: preliminary results**

Aguiar A, Jacinto AF, Teixeira SM, Ribeiro MI

Residencial Israelita Albert Einstein, São Paulo, SP, Brazil.

As Brazilian population ages, Long Term Care Institutions (LTCI) have become ideal sets for elders living under specific health conditions. Loss of daily life functions is often due to cognitive impairment related diseases. Several studies on elders’ cognitive impairment have been published and some of them have established cognitive instruments’ cutoff scores. The combination of cognitive and functional tests seems to be the effective tools in dementia diagnosis. It is possible that early detection of cognitive decline allows patients and their caregivers to plan their future more rationally.

***Objective:*** To evaluate cognitive and functional status of elders with no memory complaint living in a LTCI in the city of São Paulo, Brazil.

***Methods:*** Five elders underwent cognitive and functional evaluation. Mini-Mental State Examination (MMSE), Dementia Rating Scale (DRS) and Functional Independence Measurement (FIM) were the tools applied.

***Results:*** All of the five subjects (Table 1) got MMSE scores above cutoff (schooling was taken into consideration) and Mattis final scores under the cutoff for cognitive impairment. They were all classified as independent considering FIM scores.

***Discussion:*** The application of Dementia Rating Scale classified these subjects as probable cases of cognitive impairment whilst the other two instruments did not. DRS embraces a wider assessment of cognitive domains compared to MMSE, and that might be the reason for such results’ discrepancy. In Brazilian medical literature there was a study in which the author found a correlation between DRS scores and schooling although schooling did not seem to be the main reason for these findings differences between the tests used. DRS might be a better tool for cognitive screening in elders with Mild Cognitive Impairment. However, the time spent to be applied is longer than other tools as MMSE and FIM. Other studies with larger samples and run in different sets are necessary in order to define whether DRS is indeed a better cognitive screening instrument.

## ABSTRACT – 53

**Huntington’s disease: report of four cases among brothers**

Brum TL^1^, Costa L^2^, Brum VL^2^, Brum AV^1^

^1^Geriatric, Hospital São José do Avaí, Itaperuna, RJ, Brazil. ^2^Internal Medicine, Hospital São José do Avaí, Itaperuna, RJ, Brazil.

Huntington’s chorea, described by George Huntington in 1872, with an incidence of 5 to 10 cases per 100.000 habitants, is a degenerative, progressive disorder of the nervous system with a dominant autossomic heritage of complete penetration. There are no data in Brazil about its real prevalence or incidence. The diagnosis is performed after the observation of typical clinical manifestations (uncontrollable movements called chorea and cognitive alterations), associated to positive familiar history and complementary exams (magnetic resonance imaging of the brain, computed tomography (CT) of the brain and brain spect). The confirmation of the diagnosis should be done through molecular study by PCR. The objective of the treatment is to control the symptoms with dopamine receptor-blocking agents.

***Objective:*** To describe the presence of this disease in 4 members of a family constituted of 8 brothers.

***Results:*** ECS, male, 54 years old, married, brown, from Itaperuna/RJ, retired driver, graduated, came to the outpatient visit with his wife, referring an episode of convulsive crisis for 6 years, when he started with an anticonvulsivant therapy. He also reported a recent memory deficit and humoral liability. His wife confirmed the complaints and informed that the patient has shown choreiform movements of the face and upper members for 5 years. During the physical exam, no changes in the cardiocirculatory, respiratory and abdominal systems were noted. The patient was showing autopsychic orientation preserved, temporal disorientation, gait ataxia, dysmetria, dysdiadochokinesia, upper elastic hypertonia of the upper and lower members, patellar hiperreflexia, presence of choreiform movements of the face and members (especially in the upper members), with power and sensitiveness preserved in the four segments. MEEM 14/30, 8-points Verbal Fluency test, concrete thoughts. A very important fact in his familiar history is the presence of Huntington’s disease in 3 brothers (from 7 of the total brothers) with a confirmed molecular diagnosis. The CT of the brain showed right temporal atrophy, the brain perfusion SPECT showed perfusion changes in the ganglion of the compatible base with HD and brain-vascular pathology and molecular diagnosis showed repetitions 29/41. After confirming the diagnosis of Huntington’s disease, the treatment with atypical neuroleptic was started and the patient improved his choreiform movements. Besides, the patients were submitted to cognitive rehabilitation, which allowed social reintegration of each one of them.

***Conclusion:*** Considered the most common hereditary neurovegetative disease and with a progressive evolution, it is very important to optimize the diagnosis, conduct and genetic of the patients.

## ABSTRACT – 54

**Physical comorbility in elderly psychiatric inpatients in a Psychiatric Unit of General Hospital**

Loureiro S, Ganança L, Carmo A, Ralas A, Roldão-Vieira C, Camara-Pestana L

Psychiatry Hospital Santa Maria, Lisbon, Portugal.

The aim of this study was to determine the relationship between somatic diseases in a population of acute psychiatric patients admitted in a general hospital.

***Patients and Methods:*** Fifty subjects between 65 and 84 years of age were studied. Ten men and forty women, 96% retired, 54% live alone, the main reason to admission was affective disorders.

***Results:*** All patients have at least one diagnosis of medical condition. Hypertension, urinary tract pathology, neurological disorders, lung disease and dyslipidemia were the most prevalent diseases. 58% of patients needed the collaboration of other medical specialties in global treatment mainly, cardiology, internal medicine and orthopedics. The mean global inpatient time of the elderly patients was 25,7 days. Patients with co-morbidity who need medical intervention had longer inpatient time (27.6 days) in comparison to 23.0 days for patients without co-morbidity.

***Conclusions:*** It is necessary to keep in mind the presence of physical co-morbidity in psychiatric elderly inpatients and its implication in the therapeutical planification. Early medical screening by internal medicine can also be a major factor to the optimization of the care and prevent the extension of confinement.

## ABSTRACT – 55

Examining the impact on adult children assuming or reassuming care for elderly parents with long-standing mental illness

G Kuruvilla, F Linda, A, Jennifer

Aged Persons Mental Health, Peter James Centre, Eastern Health, Forest Hill, VIC, Australia.

This research contrast the experiences of long-term carers of elderly parents who have had a long-standing mental illness and first-time carers of parents who have become mentally unwell or in need of care in old age. It was hypothesised that the children of elderly parents with long-standing mental illness have differing needs when faced with the caring role compared to first-time carers of elderly mentally ill patients. Eight carers were interviewed using a standardised questionnaire from which salient issues were drawn and analysed. Long term carers faced issues including a longer duration of care-giver stress, early entry into adult responsibilities of caring, frustrations concerning lack of recognition from mental health services and cumulative stress affecting long-term carers significant relationships and own health. First-time carers sought earlier access to mental health services, specialist information about mental health issues and coping strategies for the care-giving role.

## ABSTRACT – 56

**Depression training program for caregivers of elderly care recipients**

Kuruvilla G^1,2^, David M^2^, Sarah R^2^, McCabe Marita P^2^, Tanya DE^2^

^1^Aged Persons Mental Health, Peter James Centre, Eastern Health, Forest Hill, VIC, Australia. ^2^Psychology, Deakin University, Burwood, VIC, Australia.

The prevalence of untreated depression is high among older adults who receive care in residential facilities or in their own homes and is associated with reduced quality of life and other medical conditions. Research has suggested a number of reasons for the low detection and treatment rates for this problem, including lack of knowledge and efficacy among those who provide direct care and poor communication between these caregivers and senior staff, and between senior staff and general practitioners. In this study, we report on the implementation of a training program for care staff that aims to address these issues. Focus groups with participants who completed the training indicated a high level of satisfaction with the program and reported improvements in knowledge, self-efficacy, and communication within services.

## ABSTRACT – 57

**Comparing multifactorial memory training and psychosocial intervention in persons with MCI and healthy older adults in a double-blind randomized control design**

Gilbert B^1^, Belleville S^2^, Fontaine F^1^, Ducharme F^3^, Trudeau D^4^, Gauthier S^5^

^1^Neuropsychologie, IUGM, Montreal, QC, Canada. ^2^Psychologie, UdeM, Montreal, QC, Canada. ^3^Sciences infirmières, UdeM, Montreal, QC, Canada. ^4^Soins infirmiers, IUGM, Montreal, QC, Canada. ^5^Psychiatrie, de neurologie et neurochirurgie et de médecine, Université McGill, Montreal, QC, Canada.

Mild Cognitive Impairment (MCI) refers to older persons with impaired performance on memory tasks but who do not meet the criteria for dementia. Our previous study (Belleville et al. , 2006) has shown that multifactorial memory training (MEMO program) had a positive impact on memory performance compared to a group with no intervention. However, other factors, such as being in a group (peer effect), could account for the positive results obtained. The main goal of this study was to assess whether the significant results obtained were the effect of the memory training program in a design that controlled this factor. To do this, we compared the multifactorial memory training to a psychosocial intervention. Twenty MCI and 20 healthy older adults took part in the study. Participants were randomly assigned to memory training or psychosocial intervention. Both interventions included 7 weekly sessions of about 2 hours. The MEMO program included learning, through tutoring and observation, episodic memory strategies (method of loci, face-name association and a method of organizing text information). Psychosocial training focused on stress management, problem-solving and reframing. Pre-post intervention measures included memory tasks (face-name recall, word list learning and text recall) and different psychosocial measures. Results indicated a significant effect of the multifactorial memory training on face-name recall and of the delayed word list learning for both MCI and healthy older adults. No such effects seen with the psychosocial intervention for either the MCI or the healthy older adults. Both interventions resulted in some positive effect on the psychosocial measures particularly for healthy older adults. In summary, the results indicate that a multifactorial memory intervention improves memory performance in both normal and MCI and that the effect is not related to a mere effect of social contact and attention.

## ABSTRACT – 58

**Nursing consultation for Alzheimer’s disease patients caregivers: one year experience**

Giro AJ, Estrela A

Psicogeriatria, Hospital de Magalhães Lemos, Porto, Porto, Portugal.

Alzheimer’s disease patients caregivers burnout is expected just from the beginning of illness process. That’s why give them information about disease and its development as well as to teach them how to deal with all the changes in patients behavior is part of the program of our Service for patients with Alzheimer’s disease. Nursing consultation for caregivers is where caregivers can learn more about Alzheimer’s disease and its consequences on daily living for the patient and family. They can also learn what to do and how to do it in order to protect and stimulate them. Nursing consultation is also the site where caregivers can ask questions and where they can talk about their fears. Our first objective was to study the profile (caregiver’s demographic characteristics and satisfaction with life) and the care burden in caregivers of patients with Alzheimer’s disease. The second objective was to evaluate caregiver’s satisfaction with nursing consultation and its impact: if it was useful and helpful for them. The caregiver’s profile was assessed by a non-structured questionnaire and Satisfaction With Life Scale (SWLS) (Diener et al. , 1985) and the care burden was evaluated by Zarit Burden Interview (Zarit, 1983). Patients were moreover assessed with Barthel Index (Barthel, 1965) and the Lawton and Brody’s Index (Lawton, 1969). Functional scales were applied to patients or to caregivers during an interview.

## ABSTRACT – 59

**Community nurses’ referrals of older adults to emergency departments – a follow-up study in a Swedish context**

Kihlgren A^1^, Mamhidir A^2^, Wimo A^3^

^1^Health Academy, Örebro University, Örebro, Sweden. ^2^Department of Neurobiology, Caring Sciences and Society, Karolinska Institute, Stockholm, Sweden. ^3^KI-Alzheimer’s disease Research Center (KI-ADRC), Karolinska Institutet, Stockholm, Sweden.

Older adults living in nursing homes have complex medical conditions and are commonly referred to emergency departments. Community nurses are to ensure that older adults receive proper care in nursing homes, and when needed they are to arrange hospital referrals for residents. The present aim was study to what extent nursing home patients aged 75 or older, referred to hospitals by community nurses, utilize the emergency department over a one-year period and for what reason. A further aim was to identify factors that may explain these referrals. A cross-sectional follow-up study, examining older adults’ disabilities, resources and needs, was carried out in ten communities in Sweden. Assessments were made using RAI/MDS, version 2, among 719 patients in 24 nursing homes. During the study period, the community nurses’ views on the medical problems that caused the referral to an emergency department were followed. Data were derived from both the RAI/MDS assessments and the referral nurses’ documentation. The result shows that out of 719 residents, 209 accounted for 314 referrals to an emergency department over a one-year period. No gender differences were observed. The main reasons for referrals were falls (22%), cardiovascular problems (16%), gastrointestinal problems (12%), and infections (11%). Most of the referrals (65%) were made on weekdays during daytime hours. In 62% of the cases, there had been some kind of consultation with a physician prior to the referral. We can conclude that older adults living in nursing homes are commonly referred to emergency departments. It is important to conduct more in-depth analyses of nurses’ decision-making process and documentation leading to referrals.

## ABSTRACT – 60

**Evaluation of language in the elderly: an alternative proposal**

Marcolino J^1^, Fonseca SC^2^, Lier-De VF^2^

^1^Fonoaudiologia, UNICENTRO, Irati, PR, Brazil. ^2^Linguística, PUC-SP, São Paulo, SP, Brazil.

The Brazilian Academy of Neurology recommends tests for the diagnosis of Alzheimer’s disease. These examinations are: mini-mental examination, assessment of memory; assessment of attention, evaluation of language, executive functions, concept and abstraction; constructive skills. The majority of tests for evaluation of language don’t contemplate the variety of symptoms, leaving out the singularity and language. It is through speech or writing that the other functions (memory, attention, cognitive skills) are assessed. The difficulty linguistic infers a cognitive issue. We have, therefore, a reduction of the concept of language, which appears as “function – instrumental and representative” – of another order, whether psychological or social. This paper discusses an alternative proposal for evaluation of language with elderly that articulates language-subject. The clinician this perspective must answer the some questions to evaluate: (a) what the characteristics of speech and writing of the patient? (b) what is the effect of the speech therapist in patient? (c) what is the effect of the speech therapist in the patient’s speech? These effects characterize individual treatment. Patient P. , 76 years old, found speech therapy with difficulties of memory. She feels weakened after treatment for larynx cancer. The evaluation of language shows symptoms, old age and complaints.

## ABSTRACT – 61

**Knowledge about Alzheimer’s disease of elderly sample of community dwelling in Santos – SP**

Matioli MN, Etzel A, Nasser AM, Abrahão C, Kumagai L, Cury MR, Barros PP, Shiraiashi T, Haddad V, Soares A M

Geriatric, Lusíada University School of Medicine, Santos, SP, Brazil.

***Background:*** Alzheimer’s disease (AD) is the most cause of dementia in the elderly people, its prevalence increases with age, early diagnosis is very important to the treatment and evolution of this disease.

***Methods:*** 944 literate subjects with 60 years of age or older have been interviewed randomly during a week of Alzheimer’s disease in Santos – SP. The interview took place at beaches, SESC, shopping centers and third age balls. The questionnaire was applied by undergraduate students of medicine from Department of Geriatrics, Lusíada University School of Medicine – UNILUS, Santos (SP). The questionnaire was made of 8 questions with “yes” or “no” as answers was the following: 1) Do you have someone in your family with AD? 2) Have you ever heard about AD? 3) Do you know what AD is? 4) Do you think that forgetting frequently is part of normal aging? 5) Do you have any problem with your memory? 6) Have you ever looked for a doctor to evaluate your memory? 7) Have you ever taken any medicine for memory? 8) Do you know AD has treatment? All subjects signed a written informed consent.

***Results:*** 994 elderly subjects were evaluated, 52% were male and 48% female, mean age 72.2 (±7.2) years old, mean of schooling 9.4 (±4.8) years. Questions which had more frequency of “no” as answers were numbers: 2 (95%), 3 (69.5%), 4 (52.8%) e 8 (69.3%). Questions which had more frequency of “no” as answers were numbers: 1 (89.9%), 5 (64.9%), 6 (77.5%) e 7 (86.8%). Concerning about AD, 15.1% of subjects had someone in their family with AD; 95% have ever heard about AD; 69.5% knew what AD is and 69.3% knew that AD has treatment. Concerning about memory problems: 52.8% considered that forgetting frequently is part of normal aging, 35.1% said to have memory problems, 77.5% have never looked for a doctor to evaluate their memories and 13.2% have never taken any medicine to treat their memory problems.

***Conclusions:*** Despite the most of subjects have high schooling and knew about AD, an important part of them think that forgetting frequently is part of normal aging and said have never looked for a doctor to evaluate their memories. There are necessities of more campaigns which can explain about the importance to elderly people to evaluate their memory as a routine method to help early diagnosis of Alzheimer’s disease.

## ABSTRACT – 62

**Influences of sociodemographic characteristics in knowledge about Alzheimer’s disease of elderly sample of community dwelling in Santos – SP**

Matioli MN, Etzel A, Nasser AM, Abrahão CM, Kumagai LM, Cury MR, Barros PP, Shiraiashi T, Haddad VM, Soares AM

Geriatric, Lusíada University School of Medicine, Santos, SP, Brazil.

***Background: ***Alzheimer’s disease (DA) is the most important cause of dementia in the elderly people, its prevalence increases with the age, with the phenomenon of aging all over the world, it has been expected an increase of the number of patients with AD. Early diagnosis of AD has extreme importance to the treatment and evolution of this disease.

***Methods:*** 944 literate subjects with 60 years of age or older have been interviewed randomly during a week of Alzheimer’s disease in Santos – SP. The interview took place at beaches, SESC, shopping center and third age balls. The questionnaire was applied by undergraduate students of medicine from Department of Geriatrics, Lusíada University School of Medicine – UNILUS, Santos (SP). The questionnaire was made of 8 questions with “yes” “or “no” as answers was the following: 1) Do you have someone in your family with AD? 2) Have you ever heard about AD? 3) Do you know what AD is? 4) Do you think that forgetting frequently is part of normal aging? 5) Do you have any problem with your memory? 6) Have you ever looked for a doctor to evaluate your memory? 7) Have you ever taken any medicine for memory? 8) Do you know AD has treatment? All subjects signed a written informed consent. Sociodemographic measures studied were: gender, age (divided into two groups: 60–70 years and >70 years), educational level (divided into two groups: 1–8 years and >8 years of schooling). Bivariable statistical analysis was applied and data were interpreted at the 5% significance level (p<0.05).

***Results:*** Gender: significant statistical differences were seen in these questions: number 1 (p=0.001), 2 (p=0.00), 3 (p=0.04), 5 (p=0.00), 6 (p=0.01) e 8 (p=0.01). Female group obtained more “yes” answers in: “have heard about AD” (n=441), “know what AD is” (n=327) and “know AD has treatment” (n=331), “have memory problems” (n=188) and “have evaluated memory by a doctor” (n=117). Educational level: subjects with >8 years of schooling answered more frequently “yes” in: “have heard about AD” (n=455; p=0.00), “know what AD is” (n=380; p=0.00) and “know AD has treatment” (n=353; p<0.00). Age: 60–70 years group had more positive answers in “know what AD is” (n=303; p=0.04) and “know AD has treatment” (n=323; p=0.00). The 70 years group than 70 years old had positive answers with significant statistical differences in: “have memory problems” (n=200; p=0.01) and “have evaluated memory by a doctor” (n=135; p=0.00).

***Conclusion:*** The sociodemographic measures which showed knowledge about AD were: female gender, 60–70 years old group and educational level over 8 years.

## ABSTRACT – 63

The effectiveness of gerontological intervention in the functional performance of a patient with diagnosis of Shy Drager interned in geriatric infirmary

Matos NT^1^, Silva FS^2^, Camara CP^3^, Pegoretti KS^4^

^1-4^UNIFESP, São Paulo, SP, Brazil.

Hospitalization is considered a great risk for the elderly, to affect negative in the levels of functional capacity, in special for the patients with neurodegenerative diagnostic as the Syndrome of Shy Drager that if characterizes for the atrophy multiple system, idiopathic orthostatic hypotension with signals of diffuse involvement of the nervous system, that involve individuals between 4 and 8 decade of life and with prevalence in the masculine sort.

***Objective:*** To quantify the effectiveness of the joint intervention of the gerontological team between occupational therapist, physiotherapist, and speech therapist in geriatrics infirmary in impact of the hospital internment in the capacity of functional of elderly patient with neurodegeneration proressiva illness.

***Drawing of the study:*** One is about a case study, that used quantitative techniques of analysis and comparison, for the use of instrument of evaluation validated of functional independence.

***Materials and Methods:*** Study of elderly patient interned of June the August of 2008 in the Infirmary of Geriatrics and Gerontology in an Hospital School of the City of São Paulo – Brazil. The joint intervention between members of the gerontological team was carried through with patient H. S. , masculine sex, 85 years, 8 years of escolaridade, bachelor, catholic, former-trader and currently pensioner, as financial income receives benefit given continued – BPC and inhabits in proper house with the sister of 76 years. Patient was admitted in infirmary with diagnostic hypothesis of delirium; rheumatoid arthritis; systemic arterial hypertension; osteoarthritis; osteoporosis; orthostatic hypotension with test tilt+; former smokers, and dependent serious and disgnostic functionary of high of hiponatremia; Syndrome of Shy Drager; auditory deficit; escabiose treated; anemia; pressure ulcers calcaneal (Degree II); depressive syndrome, induced diabetes mellitus for corticoide. The functionality was surveyed with Functional Independence Measure (MIF), applied in the admission, weekly during high internment and in the hospital one.

***Results:*** Patient presented significant increase of the level of physical performance and cognition, with bigger social participation and reduction of the oscillation of mood and behavior during the long period of interment; you evidence to oppose them literary of the decline of the functional capacities in elderly for the impact of hospitalization.

***Conclusion:*** The joint performance between occupational therapist, physiotherapist and speech therapist if showed efficient in the planning, elaboration, construction and therapeutical application in the levels of functional performance for the whitewashing, maintenance and promotion of interned functional independence in aged. The incentive of the complementation and junction of the therapeutical knowledge of the different areas of the health to the promotion of quality of life in aged becomes necessary.

## ABSTRACT – 64

**Neuropsychological and clinical psychological approaches using collage techniques for patients with Alzheimer’s disease**

Meguro M, Ishizaki J, Ishii H, Yamaguchi S, Meguro K

Geriatr Behav Neurol, Tohoku Univ Grad Sch Med, Sendai, Japan.

In Japan, the collage technique of arts has been introduced as a psychotherapeutic method in late 1980s, and has been applied to various illnesses. However, this technique has not been fully applied for dementia patients. We herein analyzed the characteristics of the collage articles made by patients with Alzheimer’s disease (AD).

***Methods:*** Twenty patients diagnosed as probable AD according to the NINCDS-ADRDA criteria were studied. They did not show visual or motor symptoms that may affect the collage performance. A drawing paper from B5 to A3 size was used, and the patients were asked to select and put several pieces they like. The pieces were cut in advance and put in a box by therapists for patients. The Mini-Mental State Examination (MMSE) and Cognitive Abilities Screening Instrument (CASI) were used for neuropsychological assessments.

***Results:*** First, we analyzed the characteristics of the form aspect with reference to their neuropsychological impairments. We found the simplification and poor organization in their collage articles, which were previously reported to be similar to drawing impairments of AD patients. The tendency was severe when the MMSE and CASI scores were more decreased, especially that of visual construction. Second, we reported the content of several typical articles. We demonstrated one patient whose themes in the serial articles were changed with related to his general behavior. We discussed the images of collage articles with reference to the disease process, especially spiritual images in the early stage and family images in the later stage.

***Comments:*** We considered that the collage technique could give new perspectives for dementia patients not only by analyzing neuropsychological findings but also by exploring message from their inner world.

## ABSTRACT – 65

**Screening for depression using GDS-15 in patients with dementia**

Melo DA, Silva KM, Vasco RF, Albuquerque EV, Melo FR, Ferreira JA, Costa RT

Geriatric Outpatient Clinic, Federal University of Alagoas, Professor Alberto Antunes University Hospital, Maceió, AL, Brazil.

Geriatric Depression Scale was developed as a good instrument to screen depression among the elderly and symptoms of depression can be present in patients with dementia.

***Objectives: ***To assess the prevalence of depression using the Geriatric Depression Scale in elderly patients with dementia.

***Methodology:*** The Geriatric Depression Scale short version (GDS-15 item) and the Mini-Mental State Examination (MMSE) were administered in 38 elderly patients with dementia who had attended the Professor Alberto Antunes University Hospital of the Federal University of Alagoas. In agreement with the original scale proposed by Yesavage et al. (1983), diagnosis of depression was suggested when the cut-off point 5 for GDS-15 is reached or exceeded. The subjects who had answered the questioners had favorable cognitive conditions to do it.

***Results:*** From the total of 38 evaluated patients, 68% were female, the average age was 72.8 years old, and 18.9% were illiterate. The majority of them had Alzheimer’s disease (47.4%) and the average of MMSE was 15.4 points. The presence of depression was indicated in 52.6% of the patients. Analysing the scores found using GDS-15, 55% indicated mild depression, 20% moderate depression and 25% severe depression.

***Conclusion:*** Most of the aged patients evaluated in this study had a suggestive diagnosis of depression, thus this is important to make a systematic screening of this prevalent alteration in the behavior of patients with dementia.

## ABSTRACT – 66

**Prevalence of alterations in the Clock Drawing Test in patients with Mild Cognitive Impairment**

Melo DA, Silva KM, Vasco RF, Albuquerque EV, Melo FR, Ferreira JA, Costa RT

Geriatric Outpatient Clinic, Federal University of Alagoas, Professor Alberto Antunes University Hospital, Maceió, AL, Brazil.

The Clock Drawing Test (CDT) is a screening test for cognitive impairment, developed to evaluate damages of the visual-space abilities, constructive praxia, visual perception and capacity of abstraction. The CDT was applied in a small sample of aged (15 elderly ones) with Mild Cognitive Impairment (MCI) who had attended a private doctor’s office of Neuropsychology Clinic.

***Objectives: ***To evaluate the prevalence of alterations in the Clock Drawing Test in patients with amnestic or multidomain MCI.

***Methodology:*** Patients were diagnosed with Mild Cognitive Impairment by means of a detailed neuropsychological evaluation. Therefore the criteria alluded by Petersen et al. (2001) had been used to detect mild cognitive alterations. The Shulman’s study was also used to interpret the CDT, and the cut-off point was 4.

***Results:*** Among 15 evaluated patients, 80% were female and the average age of the sample was 73.3 years old. About the escolarity, 46.7% had more than 11 years of schooling. The averages of Mini-Mental State Examination (MMSE) and semantic Verbal Fluency were of 25.66 points and 12 animals respectively. About the CDT, 53.3% (40% with 3 points and 6.7% with 1 point) obtained score less than 4 and 46.7% obtained the maximum score (5 points).

***Conclusion:*** The majority of the patients with MCI evaluated in this study showed changes in CDT, but these changes were slight. However, the use of an additional test with constructive visuoespacial feature is important.

## ABSTRACT – 67

**The living experience of male-spouse caregivers who care for women with dementia**

Melo G1, Moreira V2

Health Community Department, ESEL, Lisbon, Portugal. 2Nursing Management, ESEL, Lisbon, Portugal.

The women, traditionally, have assumed the care to the dependent relatives. Most of previous research on caregiving reflects predominantly the women’s experience, since the samples only recruit female caregivers or just a small number of male caregivers. However, some results indicate that the male caregivers render care differently from the female caregivers, either in the volume or in the type of care, suggesting that the gender is a variable that significantly moulds the experiences of taking care in family.

***Purposes:*** To describe the experience lived by the male caregivers (spouses) of women who suffer from dementia.

***Methodology:*** The present study uses both quantitative and qualitative methodologies. Thirteen male caregivers (spouses) of women with the clinical diagnosis of dementia who took care of their wives in familiar domicile for at least two years participated in the study. The interview was constituted by two parts: a group of structured contextual questions; and the central part, not structured, had as starting point a single subject that induced for the description of the experience of living. We performed the descriptive quantitative analysis; the qualitative analysis of content, in inductive sense, was accomplished following the next steps: reading of all the texts for the understanding of the global sense; reading for identification of the units of meaning/units of register; condensation in categories; grouping in themes; new reading with the aim to refine and verify the themes; discussion, confirmation of the themes between the two authors and verification by an independent investigator.

***Results:*** The age of the caregivers was 71.7±7.08 years, education 9.62±5.02 years and the duration of care 7.5±3.64 years. Only 2 caregivers maintained professional activity. Most (11) rendered almost all the care the patient needed in the activities of daily life. Almost all (9) were isolated in the care, not receiving instrumental help and advice or emotional support. Of the qualitative analysis of content performed three themes were extracted: previous experiences, matrimonial course and decision to care.

***Conclusion:*** This is the first study indicating that the moment of decision to take care is a fundamental element in the experience of taking care for male caregivers, and probably the biggest difference of taking care between the genders. In the intervention will be important to recognize the foundations in the decision taken, which constitutes an important element in the structure of the caregivers’ experience, for it allows to clarify and to integrate the positive and negative aspects of the experience.

## ABSTRACT – 68

Short Sense Caregiver’s Competence Questionnaire: Portuguese version for family caregivers of dementia patients

Melo G^1^, Mendonça A^2^, Vernooij-Dassen M^3^, Maroco J^4^

^1^Unidade de Investigação & Desenvolvimento Enfermagem, Escola Superior de Enfermagem de Lisboa, Universidade de Lisboa, Lisbon, Portugal. ^2^Instituto de Medicina Molecular, Faculdade de Medicina, Universidade de Lisboa, Lisbon, Portugal. ^3^Joop Michels Chair Kalorama Foundation, Radboud University Nijmegen Medical Centre,, Nijmegen, Netherlands. ^4^Unidade de Psicologia e Saúde, Instituto Superior de Psicologia Aplicada, Lisbon, Portugal.

Taking care of a family patient suffering from dementia is an arduous experience. Thus, it is important, in clinical practice, to develop strategies to support relatives involved in caregiving. The objective of the Short Sense Of Caregiver’s Competence Questionnaire-SSCQ (7 items) is to assess the caregivers’ feelings of being capable of caring for a demented person. This questionnaire was based on the Sense of Competence Questionnaire, and most items were included in Zarit’s Burden interview. This instrument is useful in clinical practice due to its brevity and because the items refer to strategies for the management of caregivers’ support.

***Objective:*** The purpose of this study was to validate a Short Sense Competence Questionnaire For Portuguese family caregivers of dementia patients who live at home.

***Methods:*** The participants were 105 patients and their family caregivers, living at home, attending a Dementia Clinic in Lisbon. The translation of the SSCQ was performed by a nurse and a physician, following a discussion with other experts in the subject area, and the retroversion was done by a professional translator. Factorial validity was evaluated by exploratory factor analysis and the construct convergent validity was assessed with correlation between SSCQ and Zarit’s Burden Interview as well as average extracted by the factors. Reliability was evaluated by Cronbach’s alpha. The correlations between individual items and the total score were also calculated.

***Results:*** Caregivers: 68.6% female; age 67.0±12.5 years; 75.2% spouses and 95.2% lived together; education 7.9±4.6 years; SSCQ 23.1±6.8; Zarit’s Burden interview 31.8±14.3; CES-D 18.6±11.6. Patients: 55.2% female; age 75.4±8.1 years; education 6.3±4.4 years; mild/moderate 62.9%; Mini-Mental State Examination 13.9±7.9; Neuropsychiatric Inventory 26.4±17.1; diagnosis: Alzheimer’s disease 61.0%, Frontotemporal Dementia 17.1%, other dementias 21.9%. In our study, we identified two factors in SSCQ, who explain 63,9% of the total varience. For each factor the average variance extracted was 0.5 for both factors. The convergent construct validity was supported by the correlation between Zarit’s Burden Interview and SSCQ (r=0.71). The reliability calculated with Cronbach’s alpha was 0.80. The correlations between individual items and the total score exceeded 0.40.

***Conclusion:*** The Short Sense Of Caregiver’s Competence Questionnaire (SSCQ) is a concise instrument with satisfactory validity and reliability. The brevity of SSCQ is an important issue for clinical practice.

## ABSTRACT – 69

Quality of life and depressive symptoms in the elderly who attend and who do not attend a group interaction

Merlin MS^1^, Cozza AC^2^, Merlin SS^3^

^1^Psychology / Psychiatry, UNIARARAS / UNIFESP, Campinas, SP, Brazil. ^2^Psychology, UNIARARAS, Araras, SP, Brazil. ^3^Psychiatry, UNICAMP, Campinas, SP, Brazil.

The third age is growing lately and becomes the focus of studies which seek to determine the characteristics and needs of this population. The elderly are having many opportunities to be active socially, and in this process, no longer seeks only the diseases control, but also attention to the psychological and social well-being. This research aims to investigate and compare the presence of depressive symptoms and the quality of life in elderly people who participate in an interactive group and elderly who do not attend this group. The participants were 37 elderly attending a group interaction and 37 elderly people who do not attend to this group. The instruments used were: identification questionnaire, informed consent, the Geriatric Depression Scale (GDS) and Quality Of Life For Older Adults’ Questionnaire (WHOQOL-OLD), all applied individually and analyzed from the descriptive, correlational and comparative statistics. The results showed a higher percentage of elderly people without depressive symptoms, which emphasizes the heath characteristic of this particular sample. This result can be explained by socio-demographic data of the studied population: there was the presence of a risk factor for depression (most of the sample was female) in contrast to several protective factors of mental health as low age (lower limit for the elderly category), marital status married, high level of education and income. The quality of life of older people who attended to the interactive group was better than those who do not attend to this group (p=0.026), suggesting that participation in groups of social interaction can influence positively the quality of life of older people, especially in their perception of the impact of sensory skills (p=0.001), in their ability to have personal and intimate relationships (p=0.024), and in their total quality of life (p=0.004). This study demonstrates the importance of social interaction among older adults, especially in the perception of their quality of life.

## ABSTRACT – 70

Elder abuse among the elderly assisted at an University Based Hospital in Northeast of Brazil

Moura CF^1^, Rangel CA^1^, Lima FM^1^, Petribu K^1^, Campos M^1^, Moura G^1^, Ruggio A^2^, Cambuim F^1^, Allain R^1^, Portela D^1^, Ramiro A^1^, Junqueira F^1^, Camara C^1^, De Biase A^1^, Castro H^1^

^1^Geriatria, Universidade de Pernambuco, Recife, PE, Brazil. ^2^Gerontology, Angeles Elder Abuse Forensic Center, Los Angeles, CA, USA.

***Introduction: ***The increasingly number of reports on elder abuse cases in northeast of Brazil lead the Elderly Health Care Reference Center (CRASPI) to develop a survey to investigate recurrence of abuse among its patients. Objectives: This study has two aims:1) identify socio-demographics characteristics of the population assisted by the CRASPI and 2) to identify elder abuse among its patients.

***Methods:*** This study is a mixed methods study, with data collected from the period of August to December 2008. 165 patients 60 years and older, where interviewed, two different questionnaires were used: one composed of objective questions about abuse cases and another one about socio-demographic information. Due to the nature of the interview, elderly with hearing impairment were excluded from this study.

***Results:*** Socio-demographic aspects: participants age ranged from 60 to 95 years old, with median age of 71.18 years old. 77.5% of the sample were female, 44.8% lived in Metropolitan area of Recife, being 41.8% married. Half of the sample had completed only elementary school. Elder abuse: 43.6% of the interviewees reported suffered some type of violence. 52.9% reported being victim of psychological abuse and 35.6% reported being victims of financial abuse.

***Conclusion:*** The high recurrence of violence reported among elders using the CRASPI, reveals the significance of the elder abuse problem in northeast of Brazil, revealing yet the need for more investments across all levels of society in the prevention and care of this problem.

## ABSTRACT – 71

**The prevalence of thyroid dysfunction in elderly patients evaluated for cognitive complaints in CDA-IPUB-UFRJ**

Mukamal RC, França M, Almeida C, Laks J, Vaisman M

IPUB-UFRJ, Rio de Janeiro, RJ, Brazil.

It is well known that thyroid gland dysfunction is a common clinical problem associated with aging. Both hypothyroidism and hyperthyroidism are easily overlooked or misdiagnosed in elderly patients because of nonspecific or atypical presentation. Several studies on the epidemiology of thyroid dysfunction in aged people have been performed. The reported prevalence of hypothyroidism has varied from 0.9 to 17.5%, and that of hyperthyroidism from 0.5 to 6%. The prevalence of thyroid dysfunction has been found to be higher in women and, also, the prevalence of subclinical thyroid dysfunction is higher than that of overt thyroid dysfunction in both hypothyroidism and hyperthyroidism. More recently, thyroid dysfunction has emerged as a possible risk factor for irreversible dementia. Thyroid hormone has many effects on the heart and vascular system and this vascular risk factors may contribute to cognitive decline.

***Objective:*** This Cross-sectional study is a pilot study for the evaluation of the thyroid dysfunction in patients with cognitive complaints so as to establish a relationship between clinical and neuropsycological aspects of these subjects.

***Patients and Methods:*** The population of this study consisted of 61 patients aged 60 to 92, that were sent for evaluation in the CDA-UFRJ from April/2007 to August/2008. All subjects underwent a comprehensive clinical investigation including history, physical and psychiatric evaluation. Serum concentrations of TSH, FT4 and Anti-TPO were measured in all this patients.

***Results and Conclusion:*** In a total of 61 patients that were tested, 56 women and 15 men, 5 (8%) had an abnormal serum TSH concentration and 9 (15%) an elevated in ANTI-TPO levels. Of the subjects with abnormal TSH 2 patients (3%) had overt hypotireodism, 2 patients (3%) had subclinical hypothyroidism and 1 patient (2%) had subclinical hyperthyroidism. The prevalence of abnormal biochemical thyroid function reported here is in agreement with previous studies. Further studies with a larger sample size are needed to confirm this finding and to investigate the relationship between thyroid dysfunction neuroscicological and clinical findings of patients in investigation of dementia.

## ABSTRACT – 72

**Integration of a psychiatrist into a neurology department’s novel interdisciplinary memory clinic**

Nathan R, Geldmacher DS, Manning C, Thompson-Heisterman A

Neurology, University of Virginia Health System, Charlottesville, VA, USA.

The Memory and Aging Care Clinic of the Department of Neurology is unique in that all disciplines are represented ONSITE during the clinic. This results in continuous communication among the staff concerning each patient seen. We believe that by the interactions illustrated in our poster we will show how our team of neurologist, neuropsychologist, geriatric psychiatrist, geriatric/ psychiatric nurse practitioner, social worker, nurse coordinator, and Alzheimer association social worker representative are able to achieve our stated goals of comprehensive patient and family care. Details of the operation will be illustrated by the following: (1) initial referral and review of records, this would include previous neurological examinations, neuropsychological studies and neuroimaging reports or digital materials, (2) early morning case conference would discuss all new patients as well as returning previously evaluated ones, (3) initial diagnostic evaluation by clinic neurologist (4) neuropsychology appointment if needed, (5) if psychiatry is needed it may be done either following the neurological exam the same day if possible, or at a follow up visit, (6) all returning cases are reviewed and plans are made based on individual needs, (7) previously evaluated psychiatric referrals are seen by the psychiatrist along with other disciplines as needed. This same routine is followed at the noon conference for the afternoon patients, Each of the neurologists sees on average two patients in each morning and afternoon session. The follow-up patients usually number between five or six for each of these sessions. We intend to show how based on these twice daily case conferences we are able to assign appropriate disciplines to each case and then by formal and informal (curbside) interactions, achieve optimal results. Flow charts will illustrate these interactions as the twice daily team meetings direct the team based on patients’ needs previously established in the case of returning individuals. The clinic is a regional resource for these patients and their families, many of whom travel several hours to reach our location. This has resulted in our frequently having more than one professional in with the family during the examination. It has proven quite effective, for example, to have the psychiatrist and the nurse practitioner see the family together and formulate a treatment plan. At other times the psychiatrist and social worker have worked either together or sequentially on resolving potential placement situations. We have found the Alzheimer association representative helpful in pointing out resources throughout the region. In ten months the psychiatrist has evaluated forty seven patients, thirty one women and sixteen men; important summary aspects of these evaluations will be illustrated. Compilations of these clinical examples will be used to show these complex interactions and their outcomes. Finally there will be an illustration of the economic solutions that have been implemented to ensure adequate reimbursement for the services in order to sustain the model. Differences between this and usual Consultation-Liaison will be outlined.

## ABSTRACT – 73

**Cerebrovascular disease is highly prevalent in cases of dementia in Brazil: a neuropathological study**

Nitrini R^1^, Grinberg LT^2^, Ferretti RE^3^, Farfel JM^3^, Leite R^3^, Pasqualucci CA^2^, Saldiva PH^2^, Jacob W^3^, Brazilian Aging Brain Study Group^3^

^1^Neurology, University of São Paulo, São Paulo, SP, Brazil. ^2^Pathology, University of São Paulo, São Paulo, SP, Brazil. ^3^ Clinical Medicine (Geriatrics), University of São Paulo, São Paulo, SP, Brazil.

Alzheimer’s disease (AD) has been considered the most frequent cause of dementia in Latin America, although neuropathological confirmation has seldom been performed.

***Objectives:*** To investigate the causes of dementia in a Brazilian sample.

***Methods:*** Brains from individuals aged ≥50 years submitted to autopsy in the São Paulo Autopsy Service were collected in the Brain Bank of the Brazilian Aging Brain Study Group. Neuropathological examinations were carried out based on accepted criteria, using immunohistochemistry. The cognitive status was assessed through a post-mortem structured interview with an informant, which included the CDR and the IQCODE. For this study only cases with moderate or severe dementia (CDR ≥2 and IQCODE>3.41) were selected. The study was approved by the Ethics Committee and a responsible person signed a written informed consent form.

***Results:*** From April 2004 to March 2007, brains from 206 cognitively impaired individuals were collected. From these, 88 cases had moderate or severe dementia and were investigated. Twenty-six cases (29.5%) fulfilled the CERAD criteria for definite or probable AD, while altogether 43 cases (48.9%) had the neuropathological diagnosis of AD associated or not with other neuropathological changes, including cerebrovascular disease (CVD). When cases aged>75 years and with infrequent neuritic plaques were also classified as AD, 54.5% of the cases were diagnosed as AD (possible, probable or definite AD, as the only diagnosis or associated with other diseases). Vascular dementia (VaD) was pathologically diagnosed in 21 cases (23.9%), but altogether 40 cases (45.4%) had CVD associated or not with other neuropathological changes (including AD). AD plus CVD (“mixed dementia”) was diagnosed in 17 cases (19.3%).

***Conclusions:*** AD was the most frequent cause of dementia in this study, but the frequency of AD was lower whereas the frequencies of VaD and mixed dementia were higher than those reported in studies from developed countries.

## ABSTRACT – 74

**Remuneration in geriatric psychiatry in North America & impact on elder care**

Northcott CJ

Psychiatry, University of British Columbia, Vancouver, BC, Canada.

Access to mental health care services remains limited for the older population in North America and is particularly difficult for the frail and ethnic minority elderly. Demographics reveal that the “over 65’s” now constitute approximately 12% of the population with an increasing proportion having mental health concerns. Barriers to geriatric-oriented care appear to include inadequate funding and reimbursement to those providing psychiatric care. For example, US long term care facilities tend to be funded by insurance plans that do not reimburse for the longer assessment required for the multiple physical and psychiatric problems of their residents. Here, we will compare models of health insurance and psychiatrists’ remuneration in both Canada and the United States. We will examine the benefits and limitations of each system, and focus on how the quality of elder mental health care is influenced by each model.

## ABSTRACT – 75

**What factors account for age-related decline in hazard perception ability of older drivers?**

Horswill MS^1^, Marrington SA^1^, McCullough CM^1^, Wood J^2^, Pachana NA^1^, McWilliam J^1^, Raikos M ^1^

^1^School of Psychology, University of Queensland, Brisbane, QLD, Australia. ^2^School of Optometry, Queensland University of Technology, Brisbane, QLD, Australia.

Hazard perception ability has been tied to crash risk in older adult populations. In this study a sample of 118 older Australian drivers aged 65+ completed a video-based hazard perception test and an assessment battery designed to measure aspects of cognitive ability, vision and simple reaction time as these might be linked with hazard perception ability. The data showed that hazard perception response times significantly increased with increasing age. However, it was also found that contrast sensitivity and Useful Field of View (UFOV) performance could in large part account for this age-related increase in hazard perception response times. Contrast sensitivity, UFOV and simple reaction time accounted for the variance in hazard perception, independent of one another and of individual differences in age. Implications for policy and practice are discussed.

## ABSTRACT – 76

**Improving outcomes of driving cessation for older people**

Gustafsson L^2^, Liddle J^2^, Pachana N A^1^, McKenna K^2^, Mitchell G^3^, Haynes M^4^

^1^School of Psychology, University of Queensland, Brisbane, QLD, Australia. ^2^School of Health & Rehabilitation Science, University of Queensland, Brisbane, QLD, Australia. ^3^School of Medicine, University of Queensland, Brisbane, QLD, Australia. ^4^School of Social Science, University of Queensland, Brisbane, QLD, Australia.

Driving cessation has been recognised as having substantial negative impact on the health and wellbeing of older people. The University of Queensland Driver Retirement Initiative (UQDRIVE) program was developed to improve lifestyle and quality of life of older people facing driving cessation. Based on interviews with over 250 older drivers, retired drivers, family members and health professionals, it includes a number of community based group interventions to improve mobility and maintain social interaction. A randomised controlled trial of UQDRIVE is underway in Australia, with community-dwelling adults aged 60+ who had permanently ceased driving or were planning to do. Participants were randomised to either the UQDRIVE intervention or current practice (no intervention). Measures of wellbeing and lifestyle outcomes were undertaken prior to the intervention, immediately after the intervention and three months post-intervention. For intervention participants, individual transport and lifestyle goal setting and evaluation using the Canadian Occupational Performance Measure (COPM©) were undertaken pre and post the intervention. Goals set by group participants included adjustment to driving cessation, driving safety, and role participation. Preliminary analyses of COPM scores indicate significant differences pre and post in perceived performance (mean difference=3.22 points; t=7.11; df=19; p<0.0001) and satisfaction with performance (mean difference=3.53 points; t=6.52; df=19; p<0.0001) scales. It appears this intervention enhances coping of older adults with the often daunting prospect of ceasing to drive; implications for clinical practice are provided.

## ABSTRACT – 77

**Performance of a sample of elderly people with and without complaints about memory-related conditions in a neuropsychological test**

Paula EA^1^, Bottino CM^1^, Avilla R^1^, Souza de Lúcia MC^2^, Scaff M^3^, Miotto EC^2^

^1^Clinica de Memória, PROTER - Projeto Terceira Idade, São Paulo, SP, Brazil. ^2^Psicologia, Divisão de Psicologia do ICHC FMUSP, São Paulo, SP, Brazil. ^3^Neurologia, Faculdade de Medicina da USP, São Paulo, SP, Brazil.

The accelerated aging process of the population is intimately associated with the increase of both the prevalence and the incidence of chronic diseases and the development of handicaps. Complaints about memory-related conditions are frequent in elderly people, specially when they tend to compare their current performance with when they were younger. It is important to investigate the kind of subjective memory-related complaint and the cognitive functions because of the difficulty to evoke pieces of information that doesn’t depend on memory only. Objectives: To describe the performance of a sample of elderly people with and without complaints about memory-related conditions in a neuropsychological series and to verify the kind of subjective difficulties in terms of memory they’re having.

***Methods:*** 18 elderly people, with no complaints about memory (G1) and with complaints (G2), answered a neuropsychological test, semi-structured interview, scales of geriatric depression, and of basic and instrumental everyday life activities.

***Results:*** G1 was comprised of seven women (87.50%) and one man (12.5%), with an average age of 70.88 (±4.16) years, education 9.38 (±5.50) years and personal income of 3.5 (±2.88) minimal wage a month. G2 was comprised of nine women (90%) and one man, with 72.20 (±5.63) years old in average, 9.70 (±5.10) year of education and monthly income of 4.85 (±3.66) minimal wage. The epidemiological profile of the sample reveal that the great majority (78%) of the elderly interviewed mentioned having at least one disease or chronic condition. No impairments were verified with the scales applied nor in the cognitive functions that were evaluated (intellectual, short term memory, verbal episodic and visual-spacial memory; nominal and categorized Verbal Fluency; mental flexibility; selective and sustained attention and constructive praxia). G1 and G2 obtained similar performance. Based on the answers about the memory complaints, the clinical characteristic of these difficulties, according to the perception of the participants were: five elderly noticed the memory difficulty 13 months ago and it remains the same way as when it began (stable), two elderly noticed the memory difficulty seven months ago and it has an oscillating characteristic (sometimes gets better and sometimes gets worse) as time goes by and, finally, three elderly complained about difficulties with their memories beginning 112 months ago that deteriorate as they grow older. It is important to emphasize that none of the participants were undergoing any medical treatment in order to investigate their subjective memory complaints.

***Conclusions:*** The episodic memory is prone to losses and transformations during the aging process when compared to young adults and that the subjective complaint about memory difficulties, despite being present in more than 50% of that sample, don’t have any relationship with the objective memory difficulties and/or with the other cognitive function evaluated. The increase of life expectancy and the significant number of elderly people that complain about memory difficulties make this fact impossible of being ignored, being necessary the following of the performance with tests that evaluate different cognitive functions and the risk factors, for these complaints present unique characteristics and can be related not only with beliefs (ideas and feelings) concerning the capacity of memorizing as well as with neurological and/or psychiatric conditions.

## ABSTRACT – 78

**The relationship between education and older adults memory complaints**

Paulo DV, Yassuda MS

Escola de Artes, Ciências e Humanidades, Universidade de São Paulo, São Paulo, SP, Brazil.

***Background:*** It is plausible that older adults with memory deficits may complain about memory more frequently. Memory complaints may be more frequent among older adults with lower education, due to greater vulnerability to cognitive decline.

***Objectives:*** To investigate if memory complaints vary as a function of education, and to evaluate if memory complaints are associated with cognitive performance, anxiety and depressive symptoms.

***Methods:*** 67 older adults (between 60–75 years) were divided into 3 groups: 1–4 years of education (n=23), 4–8 years (n=20), and 9 or more (n=24). Protocol included brief cognitive battery BCB (memorization of 10 pictures, Verbal Fluency Animal Category VF, Clock Drawing Test TDR), a questionnaire about frequency of forgetting, the Memory Complaint Questionnaire MAC-Q, the Beck Anxiety Inventory BAI, the Geriatric Depression Scale GDS, and the Mini-Mental State Examination MMSE.

***Results:*** Significant differences were found among the 3 groups for picture recognition, VF, CDT, and MMSE. No significant differences were found among the groups for frequency of forgetting and MAC-Q, and there was no association between complaints, cognitive performance and depressive symptoms. Complaints were associated with anxiety symptoms.

***Discussion:*** memory complaints and frequency of forgetting were not related to education, cognitive performance, or depressive symptoms, yet they were associated with anxiety symptoms.

## ABSTRACT – 79

**Quality of care: design of the mental health care monitor older adults (MEMO)**

Veerbeek M^1^, Depla M^1^, Pot AM^2^

^1^Program on Aging, Netherlands Institute of Mental Health and Addiction, Utrecht, Netherlands. ^2^Clinical Psychology, VU University, Amsterdam, Netherlands.

In order to enhance the quality of care provided, there is a growing emphasis on transparency in mental health care. In the Netherlands there is no systematic way in which quality of mental health care is measured, let alone there is information about trends. This information is essential for policymakers to see whether their policy has the expected results or that adjustments should be made. Routine Outcome Monitoring (ROM) can be used to establish the quality of treatment. Besides, ROM provides regular information to professional and client about the course and severity of symptoms during treatment, which can enhance treatment and make it more efficient. A way of enabling routine data collection is the use of an electronic system. Older adults are under exposed thus far in ROM and consumer satisfaction is not yet taken into account. Because there is no nationwide system in which quality of mental health care provided for older adults is assessed, we designed the 'Mental health care monitor older adults' (MEMO). A surveillance network of mental health care institutions is started, to routinely measure quality of mental health care provided for older adults. In order to do this, an electronic system is developed. MEMO should provide insight in 1) whether older adults profit from their treatment in terms of mental and social functioning, 2) whether older adults are satisfied with the care they receive and, 3) the type of treatment older adults get. MEMO will act over a five year period and every year the same information will be collected to give insight in trends in the quality of Dutch mental health care for older adults (MEMO Basic). Besides this basic information a specific disorder will be focused on every year to give insight in the quality of mental health care provided for this specific type of clients. The first year additional data will be collected on older adults with depression (MEMO Depression). First results of MEMO are expected December 2009.

## ABSTRACT – 80

**Nursing staff and quality of care of Dutch living-arrangements for people with dementia: the design of the Monitor Nursing Home Facilities for People with Dementia**

Willemse BM^1^, Smit D^1^, De Lange J^1^, Depla MF^1^, Pot AM^2^

^1^Program on aging, Netherlands Institute of Mental Health and Addiction, Utrecht, Utrecht, Netherlands. ^2^Clinical Psychology, Free University of Amsterdam, Amsterdam, Noord-Holland, Netherlands.

Small scale group living care is rapidly increasing in the Netherlands. This type of care refers to small groups of older people with dementia living together in a home-like environment and integrating required personal care into daily routines. To provide this type of care, new nursing home facilities are being built or existing facilities are adjusted. As a result, there is a wide range of facility types available providing nursing home care. Five types can be identified: the traditional large scale nursing home, a large nursing home where small scale care is provided, group living homes nearby the mother facility, stand-alone group living homes in the community and nursing home wards in a home for the aged. The question rises where the ‘best’ care is provided in terms of quality of life of the residents, but also in terms of affordable care. Moreover, with the eye on the expected personnel shortage, it is important to explore the determinants of the care provided by facilities organized efficiently (low staff number) and where the nursing staff is satisfied. The Monitor Nursing Home Facilities for People with Dementia is designed to answer this question. We randomly selected 150 facilities, 30 facilities per type, to participate in this monitor. In each facility a care manager was interviewed, 15 randomly selected nursing staff members were asked to fill out a questionnaire, and CNA’s were asked to fill out a questionnaire about 12 randomly selected residents. Main outcome measures included in this study are the QUALIDEM concerning the quality of life of the residents, the Leiden Quality Of Work Questionnaire and Utrecht Burnout Scale-C concerning the nursing staff, and the Consumer Quality Index, the Approaches to Dementia measure and number of physical restraints and psychofarmaca to measure quality of care. Staff occupation is measured with number and level of nursing staff. Ten nursing home facilities with a relatively low occupation of nursing staff and a relatively high quality of care, quality of life of residents and job satisfaction will be identified as best-practice. Focus groups with managers, physicians, psychologists, nursing staff and family of the residents with dementia will be held to determine the factors contributing to the efficient occupation of nursing staff and good overall quality of these nursing home facilities. A first description of the data gathered in this study will be presented.

## ABSTRACT – 81

**An audit of national guidance on structural imaging in patients with suspected dementia in a mental health trust in United Kingdom**

Prasanna A^1^, Ejaz A^2^, Patel A^2^

^1^Old Age Psychiatry, Wolverhampton Primary Care Trust, Wolverhampton, United Kingdom. ^2^Birmingham and Solihull Mental Health NHS Trust, Birmingham, United Kingdom.

In their guidelines ‘Dementia: supporting people with dementia and their carers in health and social care’ (2006) the National Institute of Clinical Excellence (NICE) state that structural imaging should be used in assessment of elderly patients with suspected dementia to exclude other cerebral pathologies and help establish subtype diagnosis. Magnetic Resonance Imaging (MRI) is preferred to assist with early diagnosis and detect subcortical vascular changes, although Computerised Tomography (CT) scanning could be used. However, a cost utility analysis (Foster 1999) indicates that imaging is most useful in under-65’s with uncertain clinical diagnosis. We set out to establish adherence to NICE guidelines on structural imaging in patients with suspected dementia referred to the older adult service in Birmingham & Solihull Mental Health Foundation Trust.

***Methods:*** In June 2008, we audited all new referrals with suspected dementia to our service between September 2007 and June 2008. Data was recorded and cross-checked with medical and electronic records. We expected to find 100% criteria to meet with the standard.

***Results:*** Of 40 referrals with suspected dementia, mean age was 79 years (range 65–93). Structural imaging to confirm diagnosis was requested in 26 cases (65%); 19 patients had CT scan of the head, 1 patient had MRI, 4 patients were awaiting scan and 1 patient refused the CT scan appointment. Diagnosis of dementia (Alzheimer’s, vascular and mixed type) was confirmed using the results of the structural imaging in 20 patients. In 15 patients diagnosis had been confirmed on clinical basis.

***Conclusion:*** For the 35% patients who were not offered structural imaging and were diagnosed with vascular dementia, a possible change in diagnostic classification following a CT scan would have allowed access to anti-dementia drugs. However, this may not be the preferred choice based on clinical opinion and cost utility. While NICE expect healthcare professionals to take their guidance fully into account, this should not override their individual responsibility to make decisions appropriate to the patient, in consultation with the patient and their carers.

## ABSTRACT – 82

**Study of subjective memory complaints in an involutive psychiatric population**

Roldão-Vieira C, Vieira O, Dias V, Ganança L, Camara-Pestana L

Psychiatry Hospital Santa Maria, Lisbon, Portugal.

Although some studies show weak relation with Subjective Memory Complaints (SMC) and Objective Memory Impairment (OMI) other studies show a high percentage with OMI, around 50%. In ambulatory psychiatric patients, memory complaints are common and frequently the physician doubts that a real impairment exists, hence the need to evaluate the objectivity of complaints. Objectives: Clarify if there is an objective cognitive impairment in persons who are in an involution age, who attend psychiatric ambulatory and have SMC.

***Methods:*** 52 ambulatory psychiatric patients were selected, age 50 years or more and persistent memory complaints. Exclusion criteria were: mental retardation, active neurological or mental illness, moderate or severe depression and severe physical illness. The patients were evaluated according to several scales: GDS to exclude moderate or severe depression, Hachinski scale, CDR, MMSE, Clock Test, SKT, WAIS and Rey Complex Figure Test. Among the 52 patients, 12 were male (12%) and 30 are married (57.7%). The mean age was 69 (minimum 50 and maximum 87). Mean schooling was 6 years (minimum 3 and maximum 16). Almost all the patients have depressive disorders, actually in remission.

***Results:*** Among the group only 15 patients (28.8%) didn’t show cognitive impairment. Mild Cognitive Impairment (MCI) was found in 28 patients (53.8%), 8 patients with mild dementia (15.4%) and one patient with moderate dementia. These figures are not consistent with those obtained with the MMSE, where we have 32 patients (61.5%) without deficit, 16 patients (30.8%) with MCI and only 4 patients (7.7%) with mild dementia. Clock test shows deficit only in 14 patients (26.9%). CDR shows 12 patients with dementia (23.1%) and the others with possible dementia. With SKT only 4 patients (7.7%) were normal.

***Discussion and Conclusions:*** Memory complaints are very frequent in ambulatory psychiatric patients, but the physician tends not to attribute clinical value, considering them as subjective. Nevertheless, when submitted to a neuropsychological battery only 28.8% revealed no impairment and 27.3% are demented. This study shows that a neuropsychological test alone does not permit make the diagnosis of MCI, which is more accurately made by a “consensus evaluation group”; even the diagnosis of mild dementia is better done with several tests. This study also shows that involutional psychiatric patients have a high risk of cognitive impairment and the memory complaints should be screened for cognitive impairment.

## ABSTRACT – 83

**Dementia in the Brazilian population: prevalence estimates for 2010–2050**

Chaimowicz F^1^

^1^Internal Medicine, Federal University of Minas Gerais, Belo Horizonte, MG, Brazil.

Changing age structure and family composition of the Brazilian population will markedly influence the occurrence of Alzheimer’s disease and the availability of caregivers. Therefore, future estimates of dementia in older age are essential for public health planning.

***Objective:*** To project the future number of cases of dementia in the Brazilian older population (60 years or more) from 2010 through 2050. Method: Dementia observed prevalence from a population-based study was converted to prevalence estimates and applied to Brazilian census bureau population projections for 2010, 2030 and 2050. In the Ribeirão Preto study (Lopes et al. , 2005. Prevalence of dementia and Alzheimer’s disease in Ribeirão Preto, Brazil: a community survey in elderly population. International Psychogeriatrics 17:210) a representative stratified random sample of those aged 60 and older from three different socio-economic classes (N=1.145) was evaluated. In this study, the Mini-Mental State Examination, Fuld Object Memory Evaluation, Informant Questionnaire on Cognitive Decline in the Elderly and Bayer Activities of Daily Living Scale were applied together with clinical interviews, laboratory tests and brain computer tomography. The mean age was 70.9 years; 63.4% were female, and 52.8% had less than 4 years of education. The observed prevalence of dementia by age group varied from 1.9% (60–64y) to 21.8% (80+y) and was 6.2% to males and 6.6% to females. Cases of Alzheimer’s disease or mixed dementia represented 60.8%. Population estimates from Brazilian Institute of Geography and Statistics, released on 2008, were based on results from the 2000 Census and 2006 National Household Sample Survey.

***Results:*** The projected number of cases of dementia in Brazil in 2010 is 1.25 million. It is expected to double by 2030 (2.71 million) and quadruple in the next 4 decades, reaching 5.21 million in 2050. In 2010, 1 in every 15 Brazilians aged 60 years or more will be afflicted with the syndrome; this proportion will grow to more than 1 in 12 by 2050. Among the whole population, prevalence of dementia will reach 0.7% in 2010, 1.3% in 2030 and 2.4% in 2050. In the same period the number of persons with dementia will increase from 0.2 million to 0.6 million among those aged 60–69 years, and 0.5 million to 1.6 million among those aged 70–79 years. The proportion of persons aged 80 years or more among older people with dementia is expected to increase from 46.2% (0.6 million) to 57.5% (3.0 million) in this period. The proportion of cases among women will remain stable around 57%. The number of persons with pure Alzheimer’s disease or mixed dementia will be approximately 0,8 million in 2010, 1.65 million in 2030 and 3.17 million in 2050.

***Conclusion:*** The similarity between the methodology and observed prevalence from the Ribeirão Preto study and other major Brazilian surveys (Bottino et al. , 2008. Dem Geriatric Cog Disord 26:291, and Herrera et al. , 2002. Alzheimer Dis Assoc Disord, 16:103) supports the reliability of these projections. Bottino et al believe that due to design effect, nonresponse during the community phase and positive and negative predictive values, estimated prevalence of dementia may almost double the observed prevalence. Many other factors may modify these projections: the growing prevalence of diabetes in Brazil and, at the other side, the increase in literacy, better control of hypertension and the development of novel therapies for the prevention of dementia. These estimates suggest that prevalence of dementia will substantially increase, as older age groups increase in size. Furthermore, owing to the rapid growth of the oldest old, which presents the higher rates of dementia, the proportion of cases among older people will change: the number of cases among those aged 80 years or more will represent a major challenge for the Brazilian health care system.

## ABSTRACT – 84

**psicoED: online alternative for caregiver’s support of elders with dementia**

Soto F^1^, Cid T^1^, Bueno Y^1^, Hornero R^2^, Gil S^1^, Brezo M^2^, Franco M^1^

^1^Fundación Intras, Zamora, Spain. ^2^Grupo de Ingeniería Biomédica, Universidad de Valladolid, Valladolid, Spain.

Due to the aging process and increasing of dependency, the families often assume a great number of the elders carers. This translates into a high burden for the caregiver as well as a great loss of free time, while requires a range of knowledge to carry the responsibility. Faced with these difficulties psychoeducation has been shown to be an intervention that improves quality of life of the caregiver and the elder. Recently, new technologies of the information and communication, particularly the Internet, are being positioned as an ideal means to develop distance interventions cost-effective, even the researches are proving that distance interventions would be equivalents to some traditional face to face therapies.

***Objectives:*** Design an alternative to overcome the difficulties of access which have the caregivers of the elderly to interventions of proven effectiveness. *Characteristics of psicoED:* psicoED is a website that contains multimedia resources, characteristics of the 2.0 web and the possibility of multi-video-conference. psicoED contains, among other things: *Access to a forum of questions answered by professionals. *Review multimedia files specially designed to meet their concerns (summaries of articles, books, videos). *Participate in a forum where families and carers share with others how to face similar situations. *Meet and talk through a multi-video-conference with a group of up to six carers and a therapist for a psychoeducative intervention. *Benefits:* psicoED allows access to an intervention of proven effectiveness without the need for move, while maintaining continuity of care and strengthening social support. Thus, the caregivers have more free time, the possibility to seek help quickly and to an innumerable amount of information provided in written format (text) or visual (video). On the other hand, enables the therapist to maintain a closer monitoring and control, with better use of time and in a much more versatile than the traditional therapy, which becomes a perfect complement to traditional interventions.

## ABSTRACT – 85

**Art therapy applied to Alzheimer’s disease: a group therapy approach**

Serra C, Epel B C

Núcleo de Neurogeriatria, Hospital da Lagoa, Rio de Janeiro, RJ, Brazil.

This poster describes an art therapy approach to group therapy with elders with Alzheimer’s disease and other forms of dementia. We reflect on several aspects of such praxis, held in a public hospital in Rio de Janeiro since 2005. Weekly art therapy sessions are held in the context of a multiprofessional framework, which includes both drug therapy and cognitive stimulation. Art therapy resources are used to stimulate the patients’ sensory and perceptive skills and memory, emphasizing their capacities and strengthening their self-esteem. That not only reinforces reality orientation and socialization but also allows them to deepen their self-awareness, contact their feelings, and express themselves in richer ways. The groups’ composition is based on the patients’ profile and disease stage; techniques are chosen according to each group’s possibilities. Each session is conducted by two art therapists, working with ten-member groups in average. The authors aim at highlighting and enhancing the credibility of using art therapy in the care of demented elders in different stages of the disease. Valuing each patient’s expressive and creative skills is to value him/her as a person, reaffirming his/her life potential. By allowing patients to deliver objective results and highlighting each person’s capacities, art therapy helps them to awaken their own creativity in the midst of all losses and limitations brought about by the disease. Allowing space for creativity, artistic expression becomes a means of promoting health and integrity, generating life and renovation where finitude used to prevail.

## ABSTRACT – 86

**Strategies of intervention non-pharmacological used for individuals with dementia and their caregivers / family members**

Silva MR, Silvestrin M, Canon M, Arakaki B, Andrade N, Bereta P, Lima C, Rodrigues CI, Tsubaki J, Novelli M M

Universidade Federal de São Paulo, Santos, SP, Brazil.

With the increase on life expectation, diseases associated with aging, like Alzheimer’s disease (AD) and other forms of dementia, have become frequent. This research had as objective to conduct a survey of Brazilian studies with non-pharmacological interventions proposals for individuals with dementia and to their caregiver/family. The search of information was accomplished using the following databases: MedLine, PubMed, Scielo and Lilacs, considering publications by Brazilian authors from 2004. Were identified 26 articles and 18 of them were accessed. The non-pharmacological treatments proposed for individuals with dementia in those articles were: environment structuring and techniques of cognitive and neuropsychological rehabilitation, like therapy as a guide to reality, reminiscence therapy, learning without mistake, technique of reduction/extension of clues, technique of increasing the time of evocation, besides nutritional and exercise counseling. In the propositions for the caregiver/family occurred predominance in the formation of psychosocial groups related with support, education, counseling and training. It is known that there is no cure for dementia yet, so it is necessary the combination of pharmacological and non-pharmacological interventions to promote a disease slowdown progression and a better quality of life for the individuals with dementia and also for the caregivers/familiars.

## ABSTRACT – 87

Differences between Frontotemporal Dementia and probable AD patients regarding the discrimination of facially conveyed emotions: a study with Signal Detection Theory

Sobral MR, Oliveira AM

Psychogeriatrics, Hospital Magalhães Lemos, Porto, Portugal. Institute of Cognitive Psychology, University of Coimbra, Coimbra, Portugal.

Frontotemporal Dementia (FTD) is a neurodegenerative disease characterized by behavioral disorders that suggest abnormalities of emotional processing. In the past few years several studies investigated the recognition of facial emotion by Frontotemporal Dementia patients (Kessels et al. , 2007, Fernandez-Duque et al. , 2005, Keane et al. , 2002, Lavenu et al. , 1999, 2005, Perry et al. , 2001, Rosen et al. , 2002, 2004). Evidence gathered converges to suggest that inability to recognize facial emotions in FTD results from inability to recognize emotions rather than from failure in recognizing facial features. The aim of this study was to examine the discrimination of facial expression of emotions in patients with FTD and to compare it with that of patients with Alzheimer’s disease (AD). One group of FTD (n=6), another of probable AD patients (n=6), and a sample of matched controls (n=6) were compared on same-different roving tasks involving the discrimination of emotion-conveying faces (of a same person) and of individual faces (same or different persons). Two different sets of stimuli were accordingly used: (1) pairs of intensities of a same emotion, with fear, sadness, and joy as the selected emotions, in the emotion task; (2) pairs of neutral faces in the non-emotion task. Patients and controls were compared on sensitivity and criteria parameters derived from Signal Detection Theory (SDT). All subjects were given beforehand the Mini-Mental State Examination (Folstein et al, 1975), the Clinical Dementia Rating (Hughes et al. , 1982) and two Activities Of Daily Life scales. Patients were moreover assessed with a battery of neuropsychological tests and with the Frontal Behavioral Inventory (Kertesz et al. , 1997). Both groups of patients exhibited a deficit in the discrimination of facial expressions of emotion regarding the controls, but not in the task involving the discrimination of different faces. This might suggest a differential pattern of sensitivity between the two groups of demented patients resting on the distinction between a positive emotion such as joy (which FTD seem to handle better) and a negative-low activation emotion such as sadness (which AD seem to handle better).

## ABSTRACT – 88

Aranda and Knight revisited: an updated model of sociocultural stress and coping

Spencer SM,^1^ McCallum T^2^

^1^Department of Health Promotion, Education, and Behavior, University of South Carolina, Columbia, SC, USA. ^2^Psychology, Case Western Reserve University, Cleveland, OH, USA.

In 1997, Aranda and Knight published a review on the role of culture and ethnicity on the Latino caregiving experience using the Sociocultural Stress and Coping Model (SSCM) as a theoretical framework. This article demonstrated the usefulness of the SSCM, which represented a pioneering first step toward the explicit acknowledgement of culture in research on caregiving. As the field evolves and our knowledge of the mechanisms underlying stress and coping increases, it becomes necessary to address the limitations of the SSCM and propose alternate approaches to account for differences in caregiving outcomes. We revisit the work conducted by Aranda and Knight 11 years later by revising the SSCM using an updated ecological approach to more comprehensively examine the role of culture in the caregiver stress and coping process. We summarize the limitations of the SSCM and suggest modifications that fall into three general categories: 1) Emphasize the role of culture over ethnicity, 2) Address the oversimplification of factors associated with ethnic disparities, and 3) Examine the context and multidimensionality of minority/majority stress and coping at the micro-, meso-, and macro-levels of analysis. We propose a new model that highlights the role of ‘cultural units’ in the development of a cultural lens of caregiving which is unique to each caregiver. The elements of this model are illustrated using the latest research on caregiving among African Americans and American Indians.

## ABSTRACT – 89

**Age differences in Phonemic Verbal Fluency**

Steiner VG^1^, Mansur LL^1^, Brucki S^2^, Nitrini R^2^

^1^Speech and Language Therapy - Neuropsychology, University of São Paulo, São Paulo, SP, Brazil. ^2^Neurology, Medical School of São Paulo University, São Paulo, SP, Brazil.

Verbal Fluency is one of the most frequently used instrument in clinics and research in batteries recommended for the detection for cognitive alteration. Phonemic Verbal Fluency (PVF) is an interesting variant for cognitive diagnosis particularly because studies indicate that they are less influenced by age. Objectives: To detect age differences in PVF in the original format /f/ - /a/- /s/; in addition, to compare the original format with the inclusion of the phoneme /p/.

***Methods:*** We examined age differences in PVF in forty-eight healthy individuals with ages ranging from 30 to 80 years.

***Results:*** There was no association between age and performance on the Verbal Fluency test, independent of the phoneme used. There was no phoneme effect in item-generation, when comparing the traditional format /f/ - /a/- /s/ and the /p/ phoneme.

***Conclusions:*** The traditional form of /f/- /a/- /s/ is interchangeable with the modified presentation. Therefore both forms may be used in clinical or research settings. PVF is a valuable approach for detecting cognitive alterations in the aged, given its stability throughout the ageing process.

## ABSTRACT – 90

**Impaired ABSTRACT - thinking may discriminate between normal ageing and vascular Mild Cognitive Impairment**

Sudo FK^1^, Alves G^1^, Alves CE^1^, Lanna ME^2^, Valente L^1^, Moreira DM^3^, Engelhardt E^2^, Laks J^1^

^1^CDA, IPUB/UFRJ, Rio de Janeiro, RJ, Brazil. ^2^Instituto de Neurologia Deolindo Couto/UFRJ, Rio de Janeiro, RJ, Brazil. 3Hospital Pró-Cardíaco, Rio de Janeiro, RJ, Brazil.

Mild Cognitive Impairment (MCI) is a condition that lies between normal ageing and dementia. Cerebrovascular Disease (CVD), namely white matter changes and infarcts, is highly associated with cognitive deficits, and can be a cause of both MCI and vascular dementia (VaD).

***Objective:*** The present a cross-section study intending to examine differences between unimpaired elderly individuals and patients with cognitive deficits and risk factors for CVD in performances on the CAMCOG subscales.

***Methods:*** The sample was composed of 61 individuals aged more than 60 years old (19 men and 42 women). Subjects underwent evaluation, including MRI scan, and were divided into 3 groups, according to cognitive and neuroimaging status: 16 were classified as controls, 20 had MCI (Petersen’s criteria) and 25 had VaD (DSM-IV and NINDS-AIREN). Both MCI and VaD individuals scored over 4 points on the Hachinski Ischemic Scale. Functional status was assessed with Pfeffer’s Functional Activities Questionnaire.

***Results:*** Significant differences in total CAMCOG scores were observed across the three groups (p<0.001). Subjects with VaD performed significantly worse than those with MCI in most CAMCOG subscales (p<0.001), except for calculation, visual and tactile perception. All subscales showed significant differences between controls and VaD (p<0.001). Performance on abstract - Thinking was the only subscale that showed significant difference between MCI and controls (p<0.001).

***Conclusions:*** The CAMCOG discriminated controls from MCI and VaD. The CAMCOG subscale ABSTRACT - Thinking may show impairment in patients with MCI comparing with controls and it can be useful as a screening tool for early diagnosis of cognitive deficits in patients with high risk factors for CVD.

## ABSTRACT – 91

**The validity of Thai version of the Montreal Cognitive Assessment**

Tangwongchai S^1^, Phanasathit M^2^, Charernboon T^2^, Akkayagorn L^1^, Hemrungrojn S^1^, Phanthumchinda K^3^, Nasreddine ZS^4^

^1^Psychiatry, Faculty of Medicine, Chulalongkorn University, Bangkok, Thailand. ^2^Psychiatry, Faculty of Medicine, Thammasat University, Phratumthani, Thailand. ^3^Neurology, Faculty of Medicine, Chulalongkorn University, Bangkok, Thailand. 4Clinical Neurosciences and Division of Geriatric Medicine, McGill University, Montreal, QC, Canada.

Mild Cognitive Impairment (MCI) is the prodrome of dementia or incipient dementia that is challenging in clinical practice. The Montreal Cognitive Assessment (MoCA) has been demonstrated to be valid and reliable for screening of MCI in various cross-cultural clinical sample. The objective of this study is to examine the validity and reliability of the Thai version of The MoCA test in screening for patients with amnestic MCI (aMCI). Method :The sample composed of 60 subjects consecutively included from the memory clinic at the university hospital, the King Chulalongkorn Memorial Hospital, in Bangkok, Thailand. 20 patients were diagnosed as aMCI with the Clinical Dementia Rating Scale stage 0.5 and 20 patients had been treated for mild Alzheimer’s disease (AD) according to NINCDS-ADRDA, DSM IV-TR criteria and CDR stage 1. 20 geriatric patients were randomly selected as normal subjects with CDR stage 0. All participants were assessed with Thai version of MMSE, MoCA, and CDR which were administered by trained psychiatrists. Written informed consents were given by the patients or authorizing caregivers. The internal consistency and criterion validity of Thai-MoCA was explored and compared with the CDR as the gold standard for diagnosis.

***Results:*** The internal consistency of Thai-MoCA test were demonstrated to have the cronbach’s alpha coefficient of 0.744. With the cut off score under 25 and 18, the sensitivity and specificity were 0.70 and 0.95 for aMCI, 0.80 and 0.95 for AD respectively. Thai-MoCA and MMSE appeared to have significantly positive correlation with Pearson correlation coefficient 0.901.

***Conclusion:*** Thai-MoCA showed a lower cut off score comparing to the original English version as was found in the report of MoCA Korean version. However, Thai-MoCA is a reliable and valid screening tool for diagnosis of aMCI in Thai clinical sample.

## ABSTRACT – 92

**Validity of the Montreal Cognitive Assessment Dutch version (MoCA-D)**

Thissen J2, de Jonghe JF2, Boelaarts L2, Bleecke MJ2, Dautzenberg PL1

^1^Geriatric Medicine, Jeroen Bosch Medical Hospital, Den Bosch, Br. , Netherlands. ^2^Geriatric Medicine, Medical Center Alkmaar, Alkmaar, Netherlands.

Mild Cognitive Impairment (MCI) patients may show ceiling effects on cognitive screening tests. The MoCA was developed to differentiate between normal age-related cognitive decline and MCI. Aim: To examine the predictive validity of the MoCA-D. Design: Cross-sectional observational study.

***Methods:*** Participants (n=155) were patients with a diagnosis of dementia (n=24), MCI outpatients (n=25), MCI sample from an epidemiological cohort (n=37) and normal controls (n=69). The MoCA-D, Mini-Mental State Examination (MMSE) and neuropsychological tests were administered. MoCA-D scores were compared across all groups.

***Results:*** Mean MoCA-D scores (SD) in dementia were 11.8 (4.5), MCI outpatients 22.9 (2.9), MCI epidemiological cohort 25.0 (2.3), and normal controls 25.0 (3.0): ANOVA F=115.6, df=3:151, p<.001. Post hoc analysis showed that dementia patients performed worse than MCI outpatients, and MCI outpatients performed worse than MCI epidemiological cohort and normal controls.

***Conclusion:*** The MoCA-D is a valid screening test of Mild Cognitive Impairment. This is the first study showing that the MoCA-D differentiates normal controls from MCI outpatients, but that it does not differentiate normal controls from epidemiological MCI.

## ABSTRACT – 93

**Burnout in caregivers of patients with cerebrovascular disease**

Valente LE, Alves G, Eduardo Oliveira Alves C, Sudo F, Elisa Lanna M, Engelhardt E, Laks J

Federal University of Rio de Janeiro, RJ, Brazil.

To assess burden of care and burnout in caregivers of patients with cerebrovascular disease (CVD).

***Methods:*** CVD patients (n=54) and their caregivers were consecutively assessed by self report instruments comprising a sociodemographic questionnaire, Maslach burnout inventory, Zarit Burden of care, Beck Depression and Anxiety Scales Patients were administered the Mini-Mental State Examination, Clinical Dementia Rating and Neuropsychiatric Inventory three dimensions of caregiver burnout were examined: emotional exhaustion, depersonalization and reduced personal accomplishment Along with burden of care, these dimensions were correlated with caregiver sociodemographic characteristics, anxious and depressive symptoms as well as to the patients behavioral, functional and cognitive variables Data analysis with SPSS statistical package version 15.0 and employed descriptive analysis, Pearson correlation and Mann-Whitney analyses A value of p<0.05 was adopted as statistically significant for any differences.

***Results:*** Burden of care correlated with all dimensions of caregiver burnout and with depression and anxiety symptoms Reduced accomplishment was the most prevalent dimension (38.8%), however did not show any association to caregiver mood symptoms emotional exhaustion was the only dimension that showed correlation (in a moderate level) with caregiver depression (r=0.571) and anxiety (r=0.579).

***Conclusions:*** Burden of care was associated to caregiver burnout, but none of them correlated with the level of dementia severity of behavior disorders was associated to high levels of emotional exhaustion and caregiver burden.

## ABSTRACT – 94

**Late-onset schizophrenia: case report**

Silva LF^1^, Viana BM^1^, Santos AC^1^, Prais HA^2^, Daker MV^1^

^1^Psychiatry Service, Hospital das Clínicas (HC-UFMG), Federal University of Minas Gerais (UFMG), Belo Horizonte, MG, Brazil. ^2^Department of Medical Science, Federal University of Ouro Preto (UFOP), Ouro Preto, MG, Brazil.

A 68-year-old single man, 3 years of schooling, pensioner was brought to our service of psychiatry of the old age by his brother he told that 6 months before, he had been a victim of a financial fraud in which he delivered his banking applications to some people who passed as healers. Before this event, he had been listening to voices that insulted him and threatened him of death. He believed that the “healers” were interlocutors of voices. He denied visual hallucinations and reported a significant worsening of the symptoms after the fraud. Since then, he started to fear leaving his house and believed that people on the street was talking and conspiring against him and that drug dealers of his neighborhood had a plan to kill him. There were no reports of previous psychiatric disorders or use of substances. He was shy and had few closer relationships throughout his life. The patient was diagnosed as paranoid schizophrenia and risperidone was prescribed at a dose of 2 mg/day. No systemic illnesses were observed at the clinical evaluation. He was independent for basic and instrumental activities of daily living and without cognitive deficits. Vision and hearing tests showed no alterations and cranial. CT presented only a mild diffuse cortical atrophy. After 40 days of medication use, there was significant improvement of delusions and hallucinations. After 6 months of remission, risperidone was reduced to 1 mg/day. After 2 years of remission, the medication was removed gradually. However, after 8 months of withdrawal, there was recurrence of persecutory delusions and auditory hallucinations. Risperidone was restarted at a dose of 2 mg/day and remission was achieved. However, the patient initiated with significant superior limbs tremors and bradykinesia. Because of the intensity of the tremors, risperidone was replaced by quetiapine 200 mg/day with remission of the parkinsonian and psychotic symptoms since then.

***Discussion:*** Late-onset schizophrenia is diagnosed when first onset of symptoms occurs after the age of 45. Conversely, some authors propose an additional diagnosis, very-late-onset schizophrenia-like psychosis, when the first onset of schizophrenia symptoms occurs after 60 years of age. The estimated prevalence of schizophrenia for individuals over 65 years of age range from 0.1% to 0.5%. Late-onset schizophrenia is considered an unusual disorder and, to our knowledge, there is no comprehensive data about incidence rate after the age of 60. An decreased male:female ratio is found, with 0.38:1 in the 66–75 year group. Sensory deficits, higher prevalence of cerebrovascular pathology, cerebral atrophy and increase of lateral ventricles size is usual in patients with schizophrenia-like symptoms in the old age. These have led schizophrenia researchers to ascribe late-onset psychoses to organic factors and, thus, an ambiguous position in relation to schizophrenia. For these patients, very-late-onset schizophrenia-like diagnosis, may be more appropriate. Schizoid and paranoid personalities have been already reported as a risk factor, however its contribution is still uncertain. Our patient had premorbid personality traits, but he did not fulfill the criteria for Schizoid Personality Disorder as he did for schizophrenia. The absence of organic and cognitive signs and symptoms makes the term late-onset schizophrenia the most appropriate diagnosis for our patient.

## ABSTRACT – 95

**Long-term effects of compositive cognitive training for community healthy elderly: one year follow-up**

Wei F^1^, Bo LC^2^, You C^1^, Yan C^1^, Yuan WW^1^

^1^Department of Psychiatry, Tongji Hospital of Tongji University, Shanghai, China. ^2^Shanghai Mental Health Center, Shanghai, China.

To evaluate the long-term effects of compositive cognitive training for community healthy elderly.

***Methods:*** All the participants were selected from one subdistrict of Putuo District, Shanghai by every 50 samples. 151 community healthy elderly who accord with the standard were collected at last. They were divided into cognitive intervention group (90 samples) and control group (61 samples) by sequence. The interventions (include reasoning, memory, et al) were conducted in 24 sessions over 12 weeks. All individuals were assessed by Neuropsychological Test Battery for Elderly (NTBE), Stroop color-word tests and a questionnaire “Shanghai health survey for the elderly (VER2006)” at baseline, follow-up and one year follow-up phase.

***Results:*** (1) Baseline: Except 3 subscales of NTBE, 1 subscale of Stroop color-word tests were higher in intervention group than in control group (p?0.05), there were no significant differences between intervention group and control group on scores of other neuropsychological test (p>0.05); (2) Comparison within group after intervention: 18 subscales of NTBE and 6 subscales of Stroop color-word tests were significantly improved in cognitive intervention group (p?0.05) subscales of NTBE were significantly improved and 1 subscale declined; 1 subscale of Stroop color-word tests were significantly improved and 2 subscales declined in control group (p?0.05); (3) Comparison between groups after intervention: 4 subscales of NTBE, 2 subscales of Stroop color-word tests in intervention group were better than control group after cognitive training (p≤0.05); (4)Comparison within group at one year follow-up: 18 subscales improved (reasoning ability, et al) and 5 subscales declined of NTBE, 5 subscales improved and 2 subscales declined of Stroop color-word tests in cognitive intervention group (p?0.05) 16 subscales improved and 2 subscale declined of NTBE, 7 subscale improved and 2 subscales declined of Stroop color-word tests in control group (p≤005); (5) Comparison between groups at one year follow-up: 3 subscales of NTBE (reasoning ability, et al) in intervention group were better than control group, 1 subscale of NTBE was less than control group (p≤0.05).

***Conclusions:*** The compositive cognitive training could improve the executive function and reasoning ability and other cognitive functions after compositive cognitive training of community healthy elderly Some improved cognitive functions could last for 1 year, especially for reasoning ability

## ABSTRACT – 96

**Efficacy of a structured system in memory training for older adults: the gradior method**

Porto JM^1^, Tobón Arbeláez C^1^, Rodríguez Pereira C^2^, Bueno Aguado Y^1^, Franco Martín M^2^

^1^Departamento de I+D, Fundación Intras, Zamora, Zamora, Spain. ^2^Servicio de Psiquiatría, Hospital Provincial de Zamora, Zamora, Spain.

Changes in demography and a greater life expectancy have increased the prevalence of cognitive impairment in the older adults. As our population ages, there is an increasing need to address these challenges and promote efforts to develop intervention methods that could maintain and improve cognitive resources. The main purpose of the investigation was to determine the effectiveness of a structured memory training program in people with Age-Associated Memory Impairment (AAMI). Eighty Spanish participants with a mean age of 68 years, who met diagnostic criteria for AMMI were enrolled. The intervention consisted of a 2-months structured memory training program of 16 sessions, where components that are critical to memory functioning were addressed: encoding storage and retrieval mnemic processes, attentional functions and relaxation techniques. Pre and post intervention assessment consisted of a standard neuropsychological evaluation with Mini-Cognitive Exam (MEC), Squire Subjective Memory Questionnaire for Subjective Memory Loss (SSMQ), Barcelona Test Revised (PIEN-R) Digit Span and Story Recall, and Wechsler Memory Scale (WMS) Paired Associate Learning Differences found remained significant for most tests (a<0.1), especially for the BT-R Story Recall Test (0.09), and the SSMQ (0.01). Results suggest that the intervention program implemented for this study had a higher impact on verbal memory and help reduce memory complaints. Prevention through a structured training program like the GRADIOR Method, could potentially help reduce or delay the progressive decline of cognition and improve mental abilities for better daily functioning and, independent-living in older adults

## ABSTRACT – 97

**Prospective study on the use of rivastigmine patch in Alzheimer’s dementia in routine clinical setting**

Nazir E, Elliott T, Mushtaq M

Services for Older People, South Staffordshire and Shropshire Health Care NHS Foundation Trust, Shrewsbury, United Kingdom.

Clinical and naturalistic case study to share personal experience of using the patch in terms of tolerability, clinical effectiveness and challenges in day to day use of the patch in early and late onset Alzheimer’s dementia. This prospective pragmatic study was conducted over a 6 months period from June 2008-November 2008.

***Methods:*** Data collected from a case series of 1st 10 patients started on rivastigmine patch in our service (9 newly diagnosed) Sample comprised of 50% each of early onset (n=5, 57–64 yrs) and late onset (n=5, 72–83 yrs) with Alzheimer Dementia Patients/Carers were interviewed in the clinics, at home and on telephone. We had planned to review patients at 0, 2, 4, 6, 8 weeks and finally at 3 months. We intended to have at least 2 assessments and a final assessment at 3 months for each patient. Outcome was assessed globally by using validated “Mini-Mental State Examination” scale for cognitive assessment, carers feedback/ input for assessment of Activities of Daily Living Summary of the results: 1 out of 5 tried on higher dose (20% of all) had a brief episode of sickness and dizziness within first 2 weeks which resolved completely within 2 weeks 1 out of 5 tried on higher dose (20% of all) experienced worsening of overall presentation. In 1 out of 10 patients the patch had to be discontinued because no benefit was noticed 8 out of 10 patients showed global improvement in cognition, behaviour, ADLs and mood.

***Discussion:*** Reasons for choosing the patch depended on factors such as patients/ carers choice, side effects, poor compliance with oral medications, apprehension about taking extra tablets and history of gastro intestinal problems. Problems with placing the patch included redness, irritation, red blotchy area and glue sticking to skin. Occasionally patches needed to be positioned with the aid of a tape. Smaller patches were fiddly and large patches were easier to handle.

***Conclusion:*** Patch has a potential for use as an alternative first line anti-dementia medication in newly diagnosed patients with Alzheimer’s disease – both early and late onset owing to low side effect profile and better effectiveness.

## ABSTRACT – 98

**The use of antipsychotics in dementia: are they being appropriately prescribed?**

Patel AR, Roked F, Omar A, Roked F, Ahmad R

Old Age Psychiatry, The Barberry, Birmingham, United Kingdom.

Behavioural and psychological symptoms of dementia (BPSD) include psychotic symptoms such as delusions and aberrant behaviours such as aggression and disinhibition. Antipsychotics are licensed to treat the psychotic symptoms of mental illness. However, they are being used off-license for the treatment of behavioural symptoms of dementia. Evidence for limiting the use of antipsychotics in patients with dementia include: antipsychotics can have an adverse effect on cognition; can prolong the QT interval, doubling mortality and risk of sudden death; olanzapine or risperidone can increase the risk of stroke 3 fold. The NICE Guidelines for the Administration of Antipsychotics in Dementia state that: 1) Antipsychotics should only be considered in the first instance for the treatment of dementia in the presence of severe non-cognitive symptoms (for example, severe or agitated behaviour causing significant distress or if there is an immediate risk of harm to the person or others); 2) Following risk-benefit analysis of treatment, there should be a full discussion with the patient and/or carers; 3) Changes in cognition should be assessed and recorded at regular intervals; 4) Target symptoms should be identified, quantified and documented; 5) Changes in target symptoms should be assessed and recorded at regular intervals; 6) Treatment should be time limited and regularly reviewed (every 3 months or according to clinical need).

***Objectives: ***To assess the appropriateness of antipsychotic prescribing in dementia, in terms indications, duration and review of use.

***Methods:*** All patients referred to one consultant at Birmingham and Solihull Mental Health Trust (BSMHT) from August 2005 to August 2007, who received a diagnosis of dementia, were identified from computer records. The medical notes were reviewed to assess which patients, if any, had been prescribed antipsychotics, the indication for prescribing and how the patients were monitored.

***Results:*** 70 patients were suitable for the audit; 50 had BPSD (71%), 14 of whom were prescribed antipsychotics (28%). The BPSD were classified using the Neuropsychiatric Inventory in delusions/hallucinations, aggressive/irritable/agitated, apathetic/disinhibition/aberrant motor behaviour. Of these 14 patients: 6 were continued on antipsychotics for less than 3 months (43%), 3 for 3–6 months (21%), 5 for greater than 6 months (36%). Cognitive function was documented and reviewed at regular intervals. Target symptoms and any changes were clearly documented in all notes. Those patients not treated with antipsychotics were managed in alternative ways.

***Conclusions:*** The results show that the majority (72%) of patients with BPSD are not treated with antipsychotics in keeping with the NICE guidelines: 1) Antipsychotic use is limited only to those patients whose behavioural symptoms are severe enough (based on clinical judgment); 2) The majority of patients were prescribed an antipsychotic for less than 3 months; 3) Cognitive function and target symptoms along with any changes, were documented and regularly reviewed; 4) Though the results show that one team in the trust were following the guidelines, the rest of the trust’s teams still need auditing. *Recommendations:* Based on our sample of patients, the team appears to be adhering to guidelines. However, we have still made a number of practical recommendations, to ensure good medical practice: 1) Document risk-benefit analysis of antipsychotics; 2) Document discussion with patients and relevant carers; 3) Ensure the latest evidenced based medicine is applied to the individual patient; 4) A flagging system which reminds clinicians to review prescriptions, symptoms and risk-benefit every 3 months; 5) To re-audit the prescription of antipsychotics every 12 months.

## ABSTRACT – 99

**Frontal lobe and religiousness: a case report of religiousness increase related to Mild Cognitive Impairment after frontal lobe lesion**

Viana BM^1^, Silva LF^1^, dos Santos AC^1^, Prais HA^2^, Teixeira AL^3^

^1^Psychiatry Service, Hospital das Clínicas (HC-UFMG), Federal University of Minas Gerais (UFMG), Belo Horizonte, MG, Brazil. ^2^Department of Medical Science, Federal University of Ouro Preto (UFOP), Ouro Preto, MG, Brazil. ^3^Department of Internal Medicine, School of Medicine, Federal University of Minas Gerais (UFMG), Belo Horizonte, MG, Brazil.

A 84 year-old woman, eight years of schooling, had a bilateral frontal lobe contusion, predominantly at the left, after a fall without loss of consciousness, seizure or motor deficits. Two weeks later she developed a markedly increase of her religiousness, Spiritism. She started to go to a spiritual center every day and reported increased relevance of religiousness experience in her life. Besides these changes, there was no other disrupted behavior or psychotic symptoms. These changes were significant and were observed by her family and by one of the authors, which was her psychodynamic psychotherapist for 4 months prior to the fall. During that time, she was not using any medication or had any previous or recent psychiatric disorderher Duke Religion Index (DRI), a self-rating religiousness scale, revealed very high religiousness and almost achieved the maximum religiousness score. She was submitted to a neuropsychological evaluation (WAIS-III, CERAD, MATTIS) which revealed mild impairment of several cognitive domains, including memory (verbal and visuospatial), attention and executive function (work memory, inhibitory control and cognitive flexibility). She presented also with mild depressive symptoms and Mirtazapine 30mg QD was prescribed. On short term follow-up, she evolved with partial improvement of her depressive symptoms but no change of her behavior or her religiousness.

***Discussion:*** Religiousness has been related with the temporal lobe based mainly on findings of symptoms of temporal lobe epilepsy. Lesions at the frontal lobe can affect initiation of complex motor behavior, attention, executive functioning, working memory, episodic memory, language, behavior, emotions and in some cases to religiousness. Religiousness can be divided in three dimensions: organizational, related to the attendance at religion services such as going to church; nonorganizational, related to praying or religious studying; and intrinsic religiousness, related to the subjective experience of religiousness and its influence in one’s life. Normal organizational religiousness may require a competent executive and physical function, while non organizational may depend on memory, semantic and language capacities intrinsic religiousness may depend on the integrity of the self: the temporally stable, trans-situational consistencies in behavior, dress, or political or religious ideology. Three are the proposed core cognitive domains of the self: semantic knowledge for abstract information about personal attributes; autobiographical memories for concrete and affective experiences; and will for motivation to maintain self-schemas. The two latter have anatomical underpinnings in the frontal lobes. There is a temporal relation of the traumatic brain injury, cognitive impairment and religiousness increase of our patient. This is coherent to literature data of frontal lobe’s cognitive functions and behavior control playing an important role in normal religiousness. However, to our knowledge, there has been no systematical investigation about this issue. Moreover, religiousness may depend on several cognitive domains and other factors not yet comprehensively understood.

## ABSTRACT – 100

**Relationship between the Clock Drawing Test and the artistic production of a patient with Alzheimer’s disease**

Zveiter MF^1^, Zveiter MS^1^, Bessa RC^3^, Zveiter TM^1^, Farinha RS^2^, Balbino L^1^, Laranjeira RB^1^, Mello MF^4^

^1^Clinica Medica/Saude Coletiva, Hospital Balbino/Uerj, Rio de Janeiro, RJ, Brazil. ^2^Medicina Interna, Hospital do Andarai, Rio de Janeiro, RJ, Brazil. ^3^Fonoaudiologia, Dignus, Rio de Janeiro, RJ, Brazil. ^4^Odontology, My Way, Rio de Janeiro, RJ, Brazil.

The objective of this scientific work is to show the involution of the artistic production of the patient in question and its relationship with the Clock Drawing Tests performed during his clinical follow up. Moreover, it will show the positive impact of the use of art therapy in patient’s cognition, when associated with increasing doses of rivastigmine. Patient JNRD, male, 80 years old, born in Curitiba, retired art designer migrated to the capital of Rio de Janeiro during his youth to study art design at the National School of Fine Arts. He was the winner of the competition to create the logo of the 1950 World Cup, held in Brazil. Besides being designer, the patient was also a poet and troubadour. The onset of symptoms suggestive of dementia occurred in 2001, but the clinical diagnosis was only established in 2003, in the sector of Neurology at the Hospital of the State Servers, where the patient received the prescribed medication (rivastigmine 4.5 mg bid) and has undergone to a screening for detection of reversible dementia. He has also undergone to a CT scan that showed changes suggestive of dementia. The patient was admitted to the Institution of Long Stay Dignus – Art of Living in June 2004, and had his first medical consultation in October 2004. In the time that the patient was under clinical follow up with our multidisciplinary team, he was regularly subjected to the following assessments: the Folstein’s Mini-Mental State Exam (MMSE), the Clock Drawing Test, the Verbal Fluency test, the Katz’s Activities of Daily Living Scale (ADL), the Lawton’s instrumental activities of daily living scale (IADL), as well as a mini nutritional assessment. He was accompanied by a speech therapist with training in art therapy, which held a cognitive stimulation exploring his artistic skills. This study shows, by comparison, the designs and the Clock Drawing Test made before and after the cognitive stimulation by the art therapist. In the first Clock Drawing Test held on 09/10/2004, before the introduction of art therapy in the process of cognitive rehabilitation, there is a peculiar result with the replacement of the numbers on the right by letters, and replacement of the numbers on the left by words, verses and neologisms, which may be related to the patient’s artistic training. In January 2005 however, after several months of cognitive stimulation, we noted an obvious improvement in the development of sentences and in the Clock Drawing Test, that this time contained the numbers in a logical sequence, even if incorrect. The patient was clinically monitored until his death in 2005, due to lung disease unrelated to the degenerative brain disease. Through this case, we were able to highlight the importance of cognitive rehabilitation focused on individual preferences, vocations and skills of the patient, and document his clinical improvement through validated tests.

